# Dental Implants Inserted in Fresh Extraction Sockets versus Healed Sites: A Systematic Review and Meta-Analysis

**DOI:** 10.3390/ma14247903

**Published:** 2021-12-20

**Authors:** Adam Ibrahim, Bruno Ramos Chrcanovic

**Affiliations:** 1 Faculty of Odontology, Malmö University, 214 21 Malmö, Sweden; adamib.1995@gmail.com; 2 Department of Prosthodontics, Faculty of Odontology, Malmö University, 214 21 Malmö, Sweden

**Keywords:** dental implant, failure, marginal bone loss, fresh extraction socket, healed site, systematic review, meta-analysis, meta-regression

## Abstract

The present review aimed to evaluate the difference of dental implant failure rates and marginal bone loss (MBL) between implants inserted in fresh extraction sockets or healed sites. Electronic search was undertaken in three databases, plus manual search of journals, including studies randomized or not. Meta-analyses were performed besides meta-regressions, in order to verify how the odds ratio (OR) and MBL were associated with follow-up time. The review included 163 publications. Altogether, there were 17,278 and 38,738 implants placed in fresh extraction sockets and healed sites, respectively. Pairwise meta-analyses showed that implants in sockets had a higher failure risk in comparison to healed sites: OR 1.349, all studies included; OR 2.070, only prospective non-RCTs; OR 2.487, only RCTs (all *p* < 0.001). The difference in implant failure between the groups was statistically significant in the maxilla (OR 1.616, *p* = 0.029), but not in the mandible (OR 2.192, *p* = 0.075). The MBL mean difference (MD) between the groups was −0.053 mm (*p* = 0.089). There was an estimated decrease of 0.003 in OR (*p* = 0.284) and an increase of 0.006 mm (*p* = 0.036) in the MBL MD between groups for every additional month of follow-up. In conclusion, implants placed in fresh extraction sockets present higher risk of failure than implants placed in healed sites.

## 1. Introduction

According to the first installation protocol for the modern dental implant, it was recommended that the implant should be surgically placed only after a period of healing of the alveolar socket after tooth extraction, so adequate remodeling and healing of the alveolar bone would occur in order to optimize osseointegration of the implant [1]. The placement of implants in the alveolar socket right after tooth extraction was established as a new surgical protocol [2]. The procedure aimed to reduce treatment time, to decrease the number of surgical sessions, to minimize the post-extraction resorption of the alveolar bone, to provide a positive psychological impact on the patient, and to have the ability to place the implant in an ideal axial position in relation to the tooth that once occupied the socket [3]. The immediate placement of implants has even been advocated into infected sites [4]. However, the technique is not without drawbacks, as the alveolar socket may present reduced amount of bone in order to provide implant primary stability [5]. Therefore, the approach is potentially risky.

The possible negative effect of the insertion of implants in fresh extraction sockets has raised some concerns about the long-term survival of dental implants installed by this approach. A previous systematic review on the subject had shed some light on the issue [6]. The results suggested that placement of implants in extraction sockets may have an influence on the implant failure rates when compared to installation in healed sites. This review was published several years ago, and since then, many more clinical studies evaluating both techniques have been published. It was therefore the aim of the present systematic review to perform an update on the subject, adding more information from additional studies.

## 2. Materials and Methods

This study followed the PRISMA 2020 Statement guidelines [7]. Register in PROSPERO was undertaken with the registration number CRD42021240677.

### 2.1. Objective

The purpose of the present study was to test the null hypothesis of no difference in the implant failure rates and marginal bone loss (MBL) for the insertion of dental implants in fresh extraction sockets compared to the insertion in healed sites, against the alternative hypothesis of a difference, based on a systematic review of the literature. The focused question was elaborated by using the PICO format (participants, interventions, comparisons, outcomes): In patients being rehabilitated with dental implants, what is the effect of placement of implants in fresh extraction sockets on the implant failure rates and MBL in comparison to placement in healed sites?

### 2.2. Search Strategies

An electronic search without time restrictions was undertaken in October 2021 in the following databases: PubMed/Medline, Web of Science, and Science Direct. The following terms were used in the search strategies:

(dental implant OR oral implant) AND (“fresh extraction socket” OR “immediate placement” OR “immediate insertion” OR “immediate implant”).

A manual search of dental implant-related journals (listed in the Supplementary Material) was performed. The reference list of the identified studies and the relevant reviews on the subject were also checked for possible additional studies.

### 2.3. Inclusion and Exclusion Criteria

Clinical human studies were included, either randomized or not, with information on implant failure rates in diabetic and in non-diabetic individuals, rehabilitated with cylindrical modern dental implants of commercially pure titanium or its alloys. Case reports, technical reports, animal and in vitro studies, and reviews papers were excluded. Studies evaluating mini-implants, zygomatic, orthodontic, zirconia, subperiosteal, or hollow implants were excluded.

### 2.4. Study Selection

The titles and abstracts of all reports identified through the electronic searches were read independently by the authors. For studies appearing to meet the inclusion criteria, or for which there were insufficient data in the title and abstract to make a clear decision, the full report was obtained. Disagreements were solved by discussion between the authors.

RefWorks Reference Management Software (version 4.6.241, Ex Libris, Jerusalem, Israel) was used in order to detect duplicate references in different electronic databases.

### 2.5. Quality Assessment

Quality assessment of the studies was executed according to the Quality Assessment Tool of the National Institutes of Health [8]. Studies of “good” quality were judged to have at least 7 points.

### 2.6. Definitions

An implant was considered a failure if presenting signs and symptoms that led to implant removal, i.e., a lost implant.

The insertion of an implant into a fresh extraction socket was defined as a dental implant that was placed in the alveolar socket immediately after a tooth was extracted from this socket. It is also called an ‘immediate implant’ in the literature.

The insertion of an implant in a healed site was defined as a dental implant that was placed in the site where a tooth was extracted, not immediately after the extraction, but after healing of the extraction socket could occur. This healing period could vary from a couple of weeks to many months, depending on the study.

### 2.7. Data Extraction

The following data were retrieved from the studies: year of publication, study design, country, study setting, number of patients, patients’ age and sex, implant healing period, failed and placed implants in each group, MBL, implant system, jaws receiving implants (maxilla and/or mandible), presence of smokers in the patients’ study group, and follow-up time. Contact with authors asking for missing data was performed.

### 2.8. Analyses

Implant failure (dichotomous) and MBL (continuous) were the outcomes evaluated. The statistical unit for the outcomes was the implant. The I^2^ statistic evaluated heterogeneity, and the inverse variance method was used for random-effects or fixed-effects model, depending on the heterogeneity. The estimates of relative effect for implant failure were expressed in odds ratio (OR) and in mean difference (MD) in millimeters for MBL. Meta-regressions were performed to verify how the OR and MBL were associated with the time of follow-up. The data were analyzed using OpenMeta[Analyst] [9]. A funnel plot (plot of effect size versus standard error) was drawn, with the software OpenMEE [10].

## 3. Results

### 3.1. Literature Search

**Figure 1:**
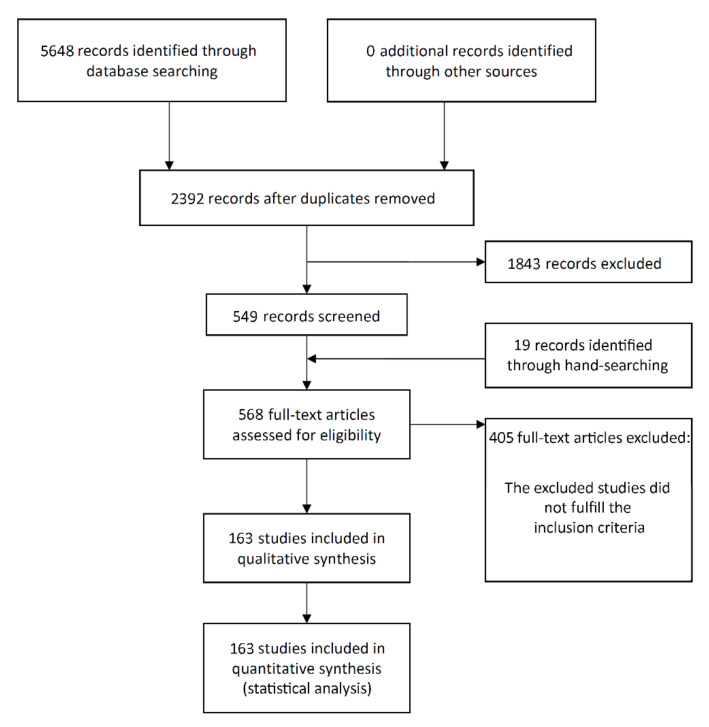
Study screening process.

The study selection process is summarized in Figure 1. The search initially resulted in 5648 papers (1626 in Pubmed, 1904 in Web of Science, 2118 in ScienceDirect), of which 163 publications were eligible for inclusion.

### 3.2. Description of the Studies

Table S1 (see Supplementary Material) presents detailed data of the 163 included studies [11,12,13,14,15,16,17,18,19,20,21,22,23,24,25,26,27,28,29,30,31,32,33,34,35,36,37,38,39,40,41,42,43,44,45,46,47,48,49,50,51,52,53,54,55,56,57,58,59,60,61,62,63,64,65,66,67,68,69,70,71,72,73,74,75,76,77,78,79,80,81,82,83,84,85,86,87,88,89,90,91,92,93,94,95,96,97,98,99,100,101,102,103,104,105,106,107,108,109,110,111,112,113,114,115,116,117,118,119,120,121,122,123,124,125,126,127,128,129,130,131,132,133,134,135,136,137,138,139,140,141,142,143,144,145,146,147,148,149,150,151,152,153,154,155,156,157,158,159,160,161,162,163,164,165,166,167,168,169,170,171,172,173]. The articles were published between 1994 and 2021. A total of 108 studies were unicenter, 50 were multicenter, and it was not possible to acquire clear information about this for the other five studies. When it comes to study design, 34 studies were randomized clinical trials (RCT), 16 were prospective studies (without a pre-established controlled group), 34 were prospective controlled clinical trials, and 79 were retrospective observational studies. For 68 studies, at least one university was reported as the institution where the study was carried out, which was the case for private dental practice for 100 studies. Multicenter studies could include the two types of institutions, namely private practices and universities. For seven studies it was not possible to acquire information on the type of institution where the study was performed. Italy was the country where the research was carried out for 68 studies (other countries could be included in case of multicenter studies). Other common places for the studies (the same observation for multicenter studies applies here) were the USA in 19 cases, Spain in 14 cases, Germany in 12 cases, Belgium in 11 cases, Brazil in 6 cases, Sweden, France, Austria, and Portugal in 5 cases each, among others.

The mean follow-up ± standard deviation of 158 studies was 34.2 ± 26.9 months (min-max, 4–124.8). For the other five studies, there was neither information on the precise time of follow-up nor the mean follow-up time. Information on follow-up in these 43 studies was usually reported as, for example, “patients were followed up between the years 2010 to 2012”, or “patients were followed up for up to 60 months”.

Immediate prosthetic loading of the implants was applied in 106 studies, early loading in 14 studies, and delayed loading in 74 studies. These loading protocols could be either separately (either immediate, or early, or delayed) applied for all implants of a study, or a combination of them for different implants of the same study. For five studies, the implants were not loaded, and for six studies, this information was not available.

Most of the studies (n = 118) included implants installed in the maxilla and mandible; 32 studies included patients that received implants only in maxillae, and the other 13 studies included only implants placed in mandibles.

Smokers were excluded from 15 studies. Information on the presence or the absence of smokers among the patients was not available for 30 studies.

Altogether, there were 17,278 implants that were placed in fresh extraction sockets and 38,738 implants placed in healed sites, and 622 and 1113 implant failures in these groups, respectively. Implants most commonly used were from the following manufacturers: Nobel Biocare (Göteborg, Sweden) in 38 studies, Straumann (Basel, Switzerland) in 19 studies, Dentsply (Mannheim, Germany) in 14 studies, and Astra Tech (Mölndal, Sweden) in 13 studies. Information on which implant brand and/or system used was not available in eight studies.

Mean MBL, separated by the focus groups of the present review, was reported in 46 studies, of which 43 also provided information on standard deviation, necessary to conduct a meta-analysis of continuous variables.

### 3.3. Quality Assessment

All included studies were classified as “good” according to the quality assessment tool (Table S2—see Supplementary Material). In most cases, the main issues in the publications were related to statistical methods not well-described and to the inclusion of non-consecutive patients in the studies.

### 3.4. Meta-Analyses

A random-effects model was used to evaluate the comparison of the implant failure between the two groups, despite the results of the I^2^ statistic (τ^2^ = 0.000, Chi^2^ = 159.915, I^2^ = 0, *p* = 0.532), as it was clearly observed that the 163 included studies presented many clinical and methodological differences. It is more clinically relevant to practitioners to identify potential sources of heterogeneity rather than simply quantify its existence. Examining for potential clinical and methodological differences between studies should be conducted rather than simply relying on tests to report the presence of heterogeneity [174]. It is important to recognize that a non-significant test for heterogeneity does not guarantee homogeneity between all trials included in a meta-analysis [175]. The I^2^ statistic has actually a relatively low power to demonstrate statistical heterogeneity, and the need to evaluate for clinical and methodological heterogeneity even in the face of little statistical heterogeneity between trials [176].

The pairwise meta-analysis showed implants placed in fresh extraction sockets had a higher risk of failure than implants placed in healed sites, with an OR 1.349 (95% CI, 1.204, 1.512, *p* < 0.001; Figure S1—Supplemental Material). An OR of 1.349 implies that implants placed in fresh extraction sockets present a 1.349 higher risk of failures happening than implants placed in healed sites; i.e., fresh extraction socket implants have a higher risk of failure by 34.9% in relation to healed site implants.

Subgroups analyses were performed for the groups of studies of different designs. The OR for implant failure when only randomized controlled trials (RCT) were pooled was 2.487 (95% CI, 1.639, 3.772, *p* < 0.001; Figure S2—Supplemental Material). The OR for implant failure when only prospective non-RCT were pooled was 2.070 (95% CI, 1.471, 2.912, *p* < 0.001; Figure S3—Supplemental Material).

**Figure 2:**
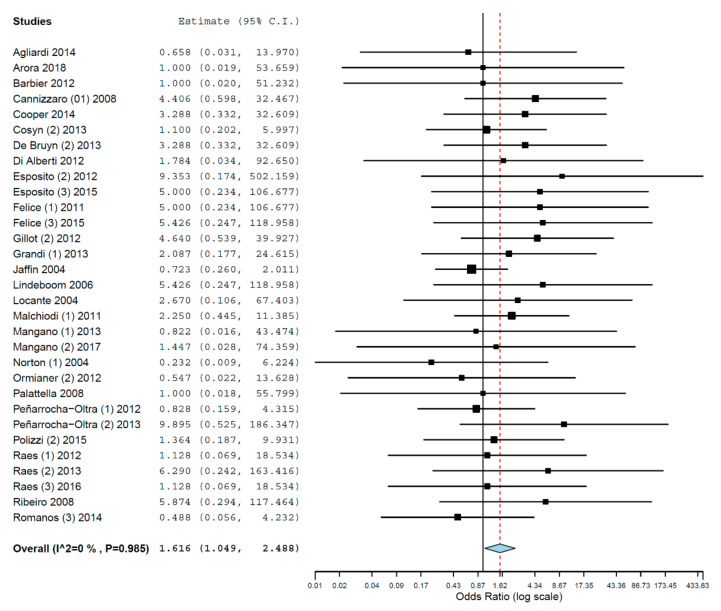
Forest plot for the event ‘implant failure’, studies evaluating implants inserted exclusively in maxillae. Estimate in Odds Ratio.

**Figure 3:**
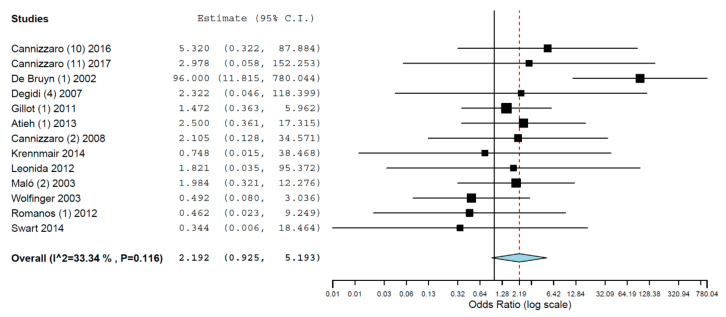
Forest plot for the event ‘implant failure’, studies evaluating implants inserted exclusively in mandibles. Estimate in Odds Ratio.

Subgroups analyses were performed for the group of studies evaluating implants inserted exclusively in different jaws. The OR for implant failure when only studies evaluating implants inserted in maxillae were pooled was 1.616 (95% CI, 1.049, 2.488, *p* = 0.029; Figure 2), and when only studies evaluating implants inserted in mandibles were pooled was 2.192 (95% CI, 0.925, 5.193, *p* = 0.075; Figure 3). Thus, the difference in implant failure between the groups was statistically significant in the maxilla, but not in the mandible.

**Figure 4:**
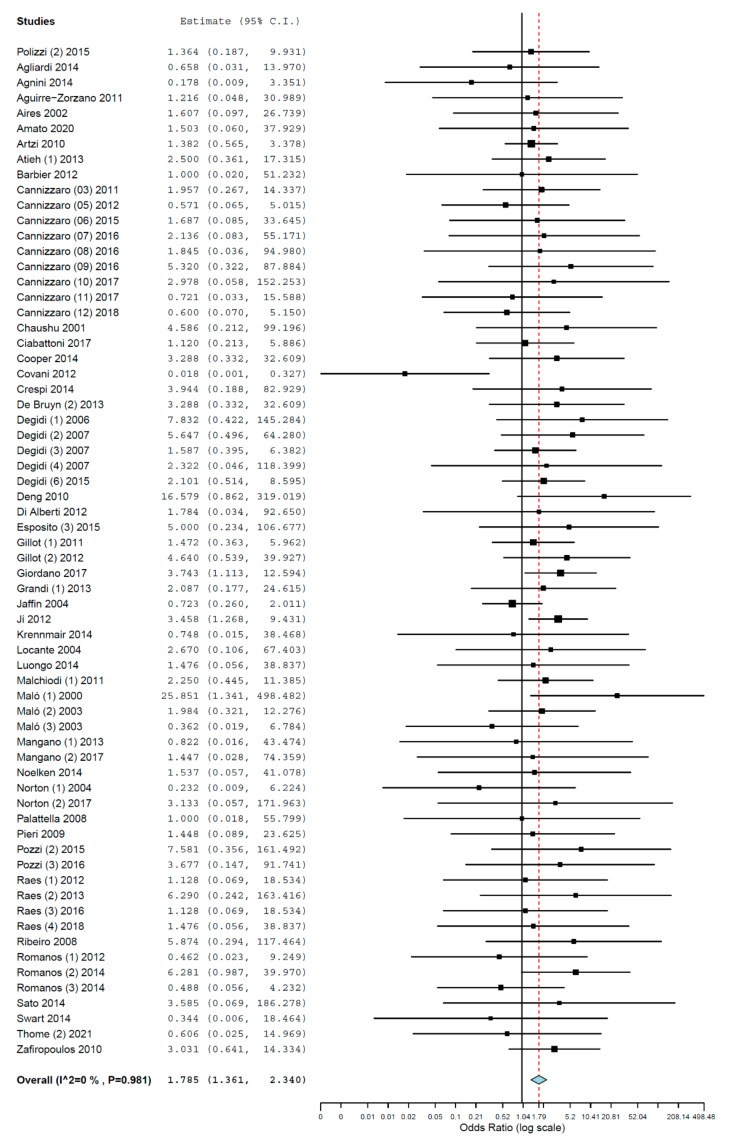
Forest plot for the event ‘implant failure’, studies evaluating implants immediately loaded exclusively. Estimate in Odds Ratio.

**Figure 5:**
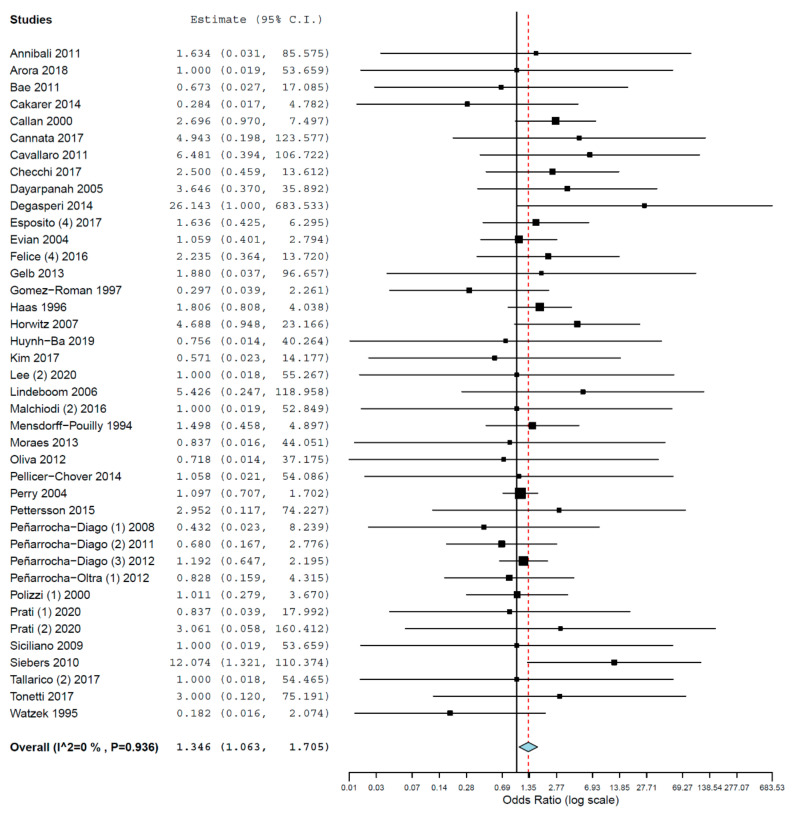
Forest plot for the event ‘implant failure’, studies evaluating implants with delayed loading exclusively. Estimate in Odds Ratio.

Subgroups analyses were performed for the group of studies evaluating implants inserted exclusively by either immediate or delayed loading protocol. The OR for implant failure when only studies evaluating implants immediately loaded were pooled was 1.785 (95% CI, 1.361, 2.340, *p* < 0.001; Figure 4), and when only studies evaluating implants with delayed loading were pooled was 1.346 (95% CI, 1.063, 1.705, *p* = 0.014; Figure 5).

**Figure 6:**
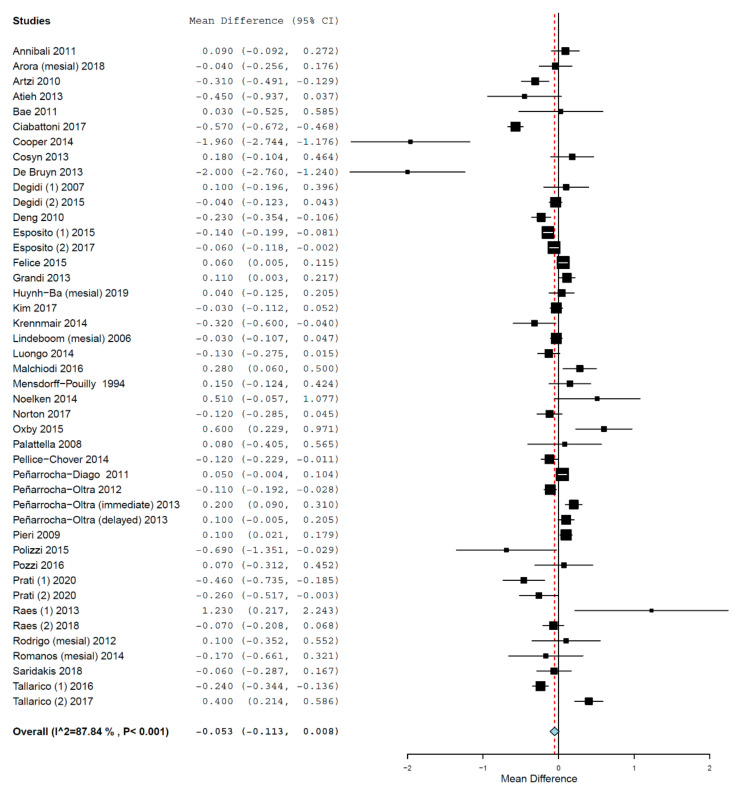
Forest plot for the event ‘marginal bone loss’.

The MD of MBL between the groups was −0.053 mm (95% CI, −0.113, 0.008, standard error 0.031, *p* = 0.089; Figure 6) (τ^2^ = 0.028, Chi^2^ = 353.522, I^2^ = 87.837, *p* < 0.001), meaning that implants placed in fresh extraction sockets presented a mean 0.053 mm higher MBL than the implants placed in healed sites. However, the difference was not statistically significant.

### 3.5. Meta-Regressions

A number of 158 studies provided clear information about the follow-up time or mean follow-up time. For the other five studies, no precise follow-up time was possible to be obtained. These studies conducted survival analysis, either life-table or Kaplan–Meier analysis, but with no mean follow-up time provided.

When a meta-regression considering the follow-up period as a covariate in relation to OR was plotted for these 158 studies, it was observed that the follow-up time did not have any effect of the OR of implant failure between the groups. The first-degree equation resulted from the linear regression of this meta-regression was

y = 0.589 − 0.003x, where:

Intercept = 0.589 (0.335, 0.843), standard error 0.130, *p* < 0.001

Follow-up = −0.003 (−0.009, 0.003), standard error 0.003, *p* = 0.284

A sensitivity analysis of the meta-regression was performed plotting together only the studies with follow-up up until 5 years. The first-degree equation resulting from the linear regression of this sensitivity analysis was

y = 0.702 − 0.008x, where:

Intercept = 0.702 (0.377, 1.028), standard error 0.166, *p* < 0.001

Follow-up = −0.008 (−0.018, 0.002), standard error 0.005, *p* = 0.132

**Figure 7:**
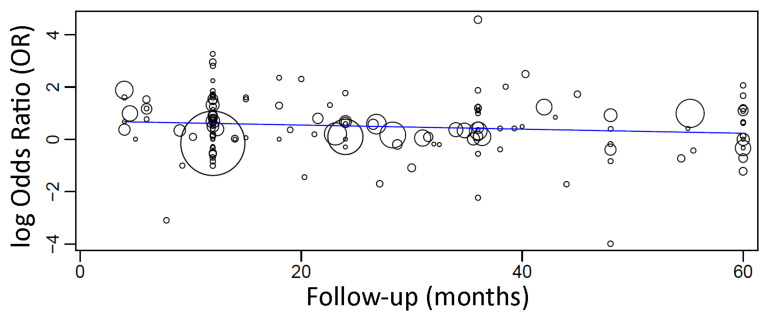
Scatter plot for the meta-regression with the association between the odds ratio (OR) of failure between implants placed in fresh extraction sockets and implants placed in healed sites, and the follow-up time (in months; limited to 60 months). Every circle represents a study, and the size of the circle represents the weight of the study in the analysis.

In this case, there was an estimated decrease of 0.008 in OR for every additional month of follow-up (Figure 7), although not statistically significant.

A meta-regression considering the follow-up period as a covariate in relation to MBL was plotted. The first-degree equation resulted from the linear regression of this meta-regression was

y = 0.001 + 0.002x, where:

Intercept = 0.001 (−0.123, 0.124), standard error 0.063, *p* = 0.993

Follow-up = 0.002 (−0.001, 0.006), standard error 0.002, *p* = 0.230

A sensitivity analysis of the meta-regression was performed plotting together only the studies with follow-up up until 5 years. The first-degree equation resulted from the linear regression of this sensitivity analysis was

y = −0.084 + 0.006x, where:

Intercept = −0.084 (−0.238, 0.071), standard error 0.079, *p* = 0.287

Follow-up = 0.006 (0.000, 0.012), standard error 0.003, *p* = 0.036

**Figure 8:**
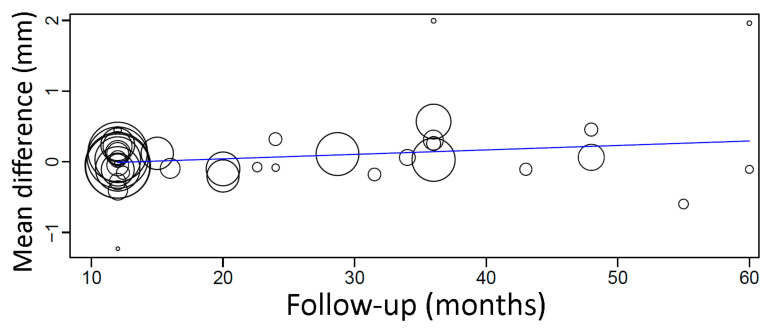
Scatter plot for the meta-regression with the association between follow-up (in months) and MBL mean difference between implants placed in fresh extraction sockets and healed sites. Every circle represents a study, and the size of the circle represents the weight of the study in the analysis.

In this case, there was an estimated increase of 0.006 mm in the mean difference of MBL between groups for every additional month of follow-up (Figure 8), being statistically significant.

### 3.6. Publication Bias

**Figure 9:**
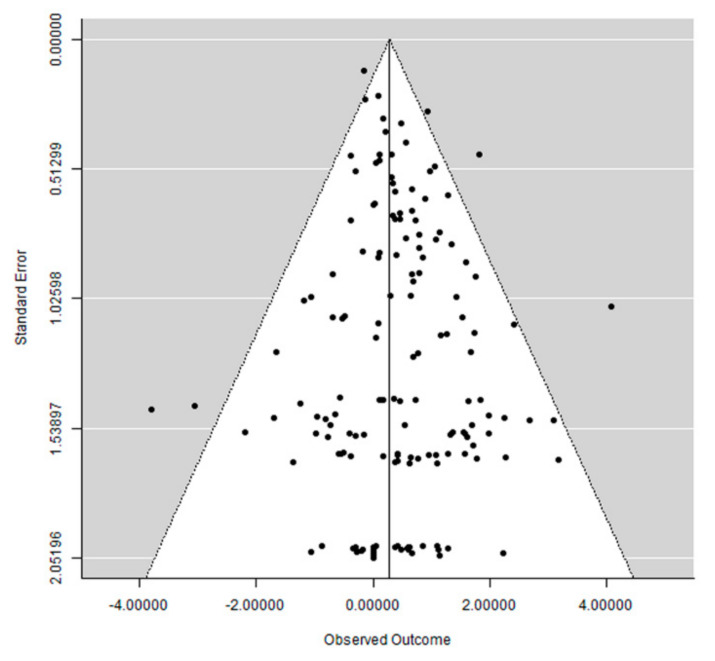
Funnel plot.

The funnel plot did not show a clear asymmetry (Figure 9), indicating possible absence of publication bias.

## 4. Discussion

According to the results of the present review, implants placed in fresh extraction sockets presented a statistically significant higher risk of failure than implants placed in healed sites. The null hypothesis regarding failure rates was therefore rejected. However, the hypothesis regarding the MBL was accepted, as the mean difference between the groups was not statistically significant.

There are some possible explanations for the higher implant failure rate when using the fresh extraction socket approach. As the extraction socket is broader than the implant in most of the cases, these implants do not usually engage all the walls of the alveolar bone, only doing so in the apical part of the socket. The primary implant stability can then be compromised [137]. Therefore, it has been recommended that this approach should only be used in the cases where there is enough apical bone to achieve primary stability of the implant, namely, the implant should engage at least 3–5 mm of apical bone [177,178]. Another option would be to install an implant that is wider than the alveolus diameter [5], but this is not feasible in many cases. Tissues affected by periodontitis may make the matters worse, as infrabony defects may result, increasing the gap between the socket walls and the immediately placed implant [179] and making it harder to achieve primary stability [180]. The occurrence of osseous defects may suggest the need of simultaneous guided bone-regeneration procedures [56], which in turn may even be helpful in increasing primary stability of implants placed immediately [181].

Subgroups analyses pooling together either only RCTs or only prospective non-RCT studies resulted in higher effect sizes compared to the analysis including all eligible studies, which included retrospective studies as well. This may be either related or not related to better study designs in relation to retrospective observational studies. The major strength of prospective studies is the accuracy of data collection regarding confounders, exposures, and endpoints [182]. Unfortunately, it was not possible to isolate confounding factors between the two groups regarding different study designs in order to verify their influence on the effect size. Moreover, the majority of the RCT studies included in the presented review were not randomized in relation to the insertion of implants in either fresh extraction sockets or healed sites. Most of these studies were randomized to other factors such as, for example, different loading protocols, number of implants supporting the prosthesis, different implant systems, different implant lengths, and use of prophylactic antibiotics.

Subgroups analyses for the group of studies evaluating implants inserted exclusively by either immediate or delayed loading protocol resulted in statistically significant odds ratio in both cases. However, the risk of failure was 78.5% and 34.6% higher for implants placed in sockets in comparison to healed sites when either immediate or delayed loading, respectively, were the exclusive protocol of choice of the studies. Only the fact that an implant is submitted to immediate loading does not lead to a lack of osseointegration and consequently fibrous tissue encapsulation, but the excessive amount of micromotion of the implant during the healing phase [183]. This may have had some influence on the fact that, in relation to implants placed in healed sites, immediately placed but delayed-loaded implants had a lower risk of failure than immediately placed and immediately loaded implants.

There was a statistically significant difference in the failure rate between the groups for implants placed in the maxilla, but not in the mandible. This could be related to the fact that sites with poorer bone quality, lack of bone volume, and thin cortical plates, which are more common in the upper jaw, may negatively affect the implant failure rates [100,184]. These characteristics of the maxilla may possibly lead to a reduction of the insertion torque of immediate implants in the upper jaw, with a consequent greater difficult-to-attain primary stability [93,185].

The results of the present study suggest that difference in MBL between the groups was not statistically significant. One might expect that the MBL around implants placed in fresh extraction sockets would be higher than in healed sites, as there is resorption of the alveolar bone after the extraction of a tooth [186]. However, there is a tendency of bone gain around an implant placed in a socket, as blood clot fills up the space between the implant and the bone walls, resulting in the formation of new bone [187], which increases in a coronal direction and finally apposes around the neck of the implant as healing takes place [145,188]. This could help to explain the reason why there was no clear difference in MBL between the approaches.

### Limitations of the Present Study

The results of the present study are not robust due to limitations. First of all, many included studies were retrospective clinical trials, which usually results in the absence of some important information in the publications. Second, many studies have a small sample size and/or a short follow-up period. The latter can result in an underestimation of number of failures. Third, several studies present a low level of specificity, meaning that their aim was not to investigate the difference in the clinical outcomes between the groups being compared in the present review. Last but not least, the studies present many confounding factors that may also affect the clinical outcomes of dental implants, not just the fact that implants were placed in fresh extraction sockets or healed sites. For example, we can cite the influence of implants of different diameters and lengths [189], status of the opposing dental arch, bruxism [190,191,192], diabetes [193], periodontal status [194], intake of different classes of medicaments by the patients [195,196,197,198], irradiation of the head and neck region [199], treatment performed by different professionals [200], different loading protocols [201], other diseases [202,203,204,205], type of prosthetic configuration [206,207,208], and patient’s sex [209], among others. Further, individual patients sometimes present with more than one risk factor [210]. The impact of these factors is difficult to estimate if these variables are not identified separately between the different groups.

## 5. Conclusions

- Implants placed in fresh extraction sockets present a 34.9% higher risk of failure than implants placed in healed sites, when results from all study designs are considered. The risk is 148.7% and 107.0% higher for implants placed in fresh extraction sockets when only randomized controlled trials (RCT) and only prospective non-RCT, respectively, were pooled together;
- The difference in implant failure between the groups was statistically significant in the maxilla (higher for fresh extraction socket implants), but not in the mandible;
- The difference in implant failure between the groups was statistically significant for either implants immediately loaded or submitted to delayed load, although the difference was higher when immediate load was applied;
- The mean difference in MBL between the groups was not statistically significant;
- There was an estimated decrease of 0.003 in odds ratio for every additional month of follow-up, although this was not statistically significant.

## Data Availability

The data presented in this study are available within the article and supplementary material.

## Supplementary Materials

The following are available online at https://www.mdpi.com/article/10.3390/ma14247903/s1, Figure S1: Forest plot for the event ‘implant failure’, Figure S2. Forest plot for the event ‘implant failure’, when only results from RCT studies were pooled together, Figure S3. Forest plot for the event ‘implant failure’, when only results from prospective non-RCT studies were pooled together, Table S1: Detailed data of the included studies, Table S2: Quality assessment tool, according to the National Institutes of Health (NIH).
