# Supplementary material for: Dental Implants Inserted in Fresh Extraction Sockets versus Healed Sites: A Systematic Review and Meta-Analysis
Source: Materials (Basel). 2021 Dec 20;14(24):7903. doi: 10.3390/ma14247903 (PMC8708389; doi:10.3390/ma14247903)
Supplement: Supplementary file 1 [file materials-14-07903-s001.zip › materials-1482678-supplementary.pdf]

SUPPLEMENTARY MATERIAL

Dental implant-related journals included in the manual search;

Figure S1. Forest plot for the event 'implant failure', global results;

Figure S2. Forest plot for the event 'implant failure', when only results from RCT studies were pooled together;

Figure S3. Forest plot for the event 'implant failure', when only results from prospective non-RCT studies were pooled together;

Table S1. Detailed data of the included studies;

Table S2. Quality assessment of the included studies, according to the National Institutes of Health (NIH).

Dental implant-related journals included in the manual search

Clinical Implant Dentistry and Related Research, Clinical Oral Implants Research, European Journal of Oral Implantology, Implant Dentistry, International Journal of Implant Dentistry, International Journal of Oral and Maxillofacial Implants, International Journal of Oral Implantology, International Journal of Prosthodontics, Journal of Clinical Periodontology, Journal of Oral Implantology, Journal of Periodontology, Journal of Prosthetic Dentistry, Journal of Prosthodontics, Journal of Prosthodontic Research.

Studies	Estimate (95% C.I.)
Agliardi 2014	0.658 (0.031, 13.970)
Agnini 2014	0.178 (0.009, 3.351)
Aguirre−Zorzano 2011	1.216 (0.048, 30.989)
Aires 2002	1.607 (0.097, 26.739)
Alves 2010	0.108 (0.005, 2.284)
Amato 2020	1.503 (0.060, 37.929)
Andersson 2015	1.410 (0.483, 4.120)
Annibali 2011	1.634 (0.031, 85.575)
Arlin 2002	0.868 (0.545, 1.384)
Arora 2018	1.000 (0.019, 53.659)
Artzi 2010	1.382 (0.565, 3.378)
Atieh (1) 2013	2.500 (0.361, 17.315)
Bae 2011	0.673 (0.027, 17.085)
Barbier 2012	1.000 (0.020, 51.232)
Bettach 2015	2.241 (0.492, 10.214)
Bogaerde 2010	10.355 (0.401, 267.097)
Cakarer 2014	0.284 (0.017, 4.782)
Callan 2000	2.696 (0.970, 7.497)
Cannata 2017	4.943 (0.198, 123.577)
Cannizzaro (01) 2008	4.406 (0.598, 32.467)
Cannizzaro (02) 2008	2.105 (0.128, 34.571)
Cannizzaro (03) 2011	1.957 (0.267, 14.337)
Cannizzaro (04) 2012	0.438 (0.020, 9.582)
Cannizzaro (05) 2012	0.571 (0.065, 5.015)
Cannizzaro (06) 2015	1.687 (0.085, 33.645)
Cannizzaro (07) 2016	2.136 (0.083, 55.171)
Cannizzaro (08) 2016	1.845 (0.036, 94.980)
Cannizzaro (09) 2016	5.320 (0.322, 87.884)
Cannizzaro (10) 2017	2.978 (0.058, 152.253)
Cannizzaro (11) 2017	0.721 (0.033, 15.588)
Cannizzaro (12) 2018	0.600 (0.070, 5.150)
Cannizzaro (13) 2018	0.438 (0.020, 9.582)
Capelli 2010	1.903 (0.074, 48.878)
Carr 2019	0.858 (0.674, 1.091)
Cavallaro 2011	6.481 (0.394, 106.722)
Cercadillo−Ibarguren 2017	0.045 (0.003, 0.783)
Chaushu 2001	4.586 (0.212, 99.196)
Checchi 2017	2.500 (0.459, 13.612)
Ciabattoni 2017	1.120 (0.213, 5.886)
Clementini 2019	1.952 (0.036, 105.522)
Cooper 2014	3.288 (0.332, 32.609)
Correia 2017	1.117 (0.457, 2.732)
Cosyn (1) 2012	0.340 (0.046, 2.513)
Cosyn (2) 2013	1.100 (0.202, 5.997)
Covani 2012	0.018 (0.001, 0.327)
Crespi 2014	3.944 (0.188, 82.929)
Cucchi 2019	1.806 (0.386, 8.445)
Dayarpanah 2005	3.646 (0.370, 35.892)
De Bruyn (1) 2002	96.000 (11.815, 780.044)
De Bruyn (2) 2013	3.288 (0.332, 32.609)
Degasperi 2014	26.143 (1.000, 683.533)
Degidi (1) 2006	7.832 (0.422, 145.284)
Degidi (2) 2007	5.647 (0.496, 64.280)
Degidi (3) 2007	1.587 (0.395, 6.382)
Degidi (4) 2007	2.322 (0.046, 118.399)
Degidi (5) 2012	0.678 (0.274, 1.677)
Degidi (6) 2015	2.101 (0.514, 8.595)
Deng 2010	16.579 (0.862, 319.019)
Dhanrajani 2005	6.286 (1.069, 36.965)
Di Alberti 2012	1.784 (0.034, 92.650)
Esposito (1) 2010	6.556 (2.675, 16.065)
Esposito (2) 2012	9.353 (0.174, 502.159)
Esposito (3) 2015	5.000 (0.234, 106.677)
Esposito (4) 2017	1.636 (0.425, 6.295)
Evian 2004	1.059 (0.401, 2.794)
Feher 2020	3.189 (0.715, 14.215)
Felice (1) 2011	5.000 (0.234, 106.677)
Felice (2) 2014	2.212 (0.193, 25.320)
Felice (3) 2015	5.426 (0.247, 118.958)
Felice (4) 2016	2.235 (0.364, 13.720)
Gehrke 2015	1.000 (0.020, 51.218)
Gelb 2013	1.880 (0.037, 96.657)
Gillot (1) 2011	1.472 (0.363, 5.962)
Gillot (2) 2012	4.640 (0.539, 39.927)
Giordano 2017	3.743 (1.113, 12.594)
Gomez−Roman 1997	0.297 (0.039, 2.261)
Grandi (1) 2013	2.087 (0.177, 24.615)
Grandi (2) 2015	1.191 (0.073, 19.570)
Guarnieri 2020	1.129 (0.440, 2.902)
Haas 1996	1.806 (0.808, 4.038)

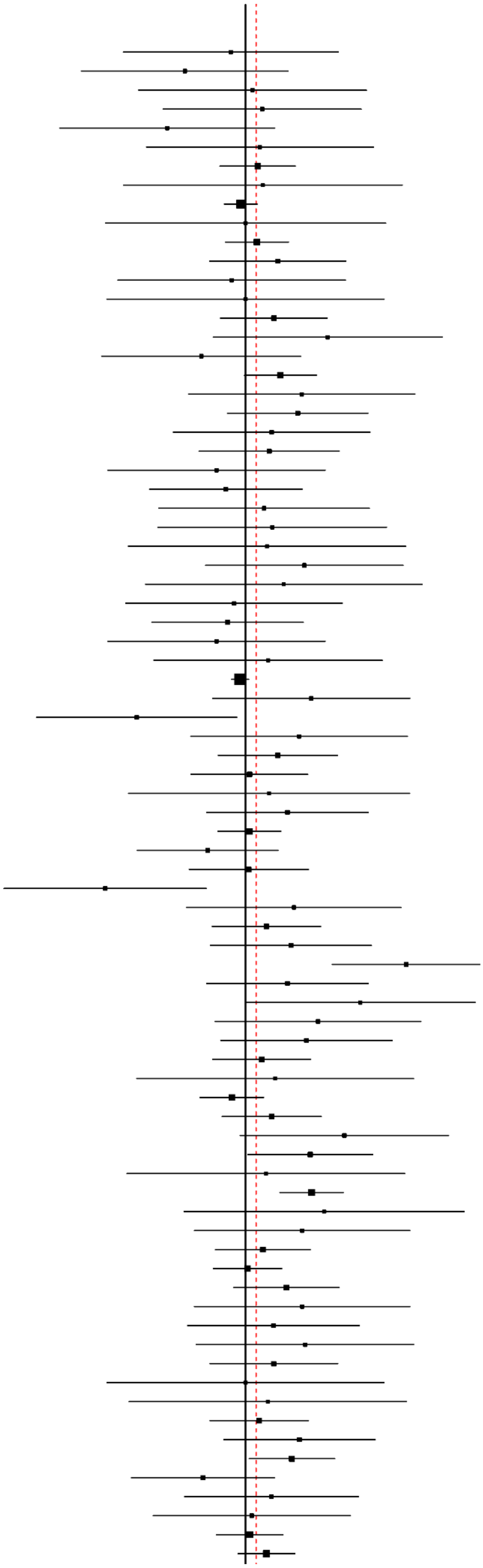

Study	OR	95% CI lower	95% CI upper
Horwitz 2007	4.688	(0.948,	23.166)
Huynh-Ba 2019	0.756	(0.014,	40.264)
Irinakis 2009	0.365	(0.017,	7.810)
Jaffin 2004	0.723	(0.260,	2.011)
Ji 2012	3.458	(1.268,	9.431)
Kim 2017	0.571	(0.023,	14.177)
Kohen 2016	1.644	(0.857,	3.153)
Kourtis 2004	2.709	(1.547,	4.742)
Krennmair 2014	0.748	(0.015,	38.468)
Lee (1) 2018	1.034	(0.288,	3.713)
Lee (2) 2020	1.000	(0.018,	55.267)
Leonida 2012	1.821	(0.035,	95.372)
Lindeboom 2006	5.426	(0.247,	118.958)
Locante 2004	2.670	(0.106,	67.403)
Luongo 2014	1.476	(0.056,	38.837)
Malchiodi (1) 2011	2.250	(0.445,	11.385)
Malchiodi (2) 2016	1.000	(0.019,	52.849)
Maló (1) 2000	25.851	(1.341,	498.482)
Maló (2) 2003	1.984	(0.321,	12.276)
Maló (3) 2003	0.362	(0.019,	6.784)
Mangano (1) 2013	0.822	(0.016,	43.474)
Mangano (2) 2017	1.447	(0.028,	74.359)
Meizi 2014	1.413	(0.359,	5.565)
Mensdorff−Pouilly 1994	1.498	(0.458,	4.897)
Mertens 2011	1.844	(0.068,	49.695)
Moraes 2013	0.837	(0.016,	44.051)
Noelken 2014	1.537	(0.057,	41.078)
Norton (1) 2004	0.232	(0.009,	6.224)
Norton (2) 2017	3.133	(0.057,	171.963)
Oliva 2012	0.718	(0.014,	37.175)
Ormianer (1) 2008	5.898	(0.605,	57.526)
Ormianer (2) 2012	0.547	(0.022,	13.628)
Oxby 2015	1.524	(0.030,	77.675)
Palattella 2008	1.000	(0.018,	55.799)
Pellicer−Chover 2014	1.058	(0.021,	54.086)
Perry 2004	1.097	(0.707,	1.702)
Pettersson 2015	2.952	(0.117,	74.227)
Peñarrocha−Diago (1) 2008	0.432	(0.023,	8.239)
Peñarrocha−Diago (2) 2011	0.680	(0.167,	2.776)
Peñarrocha−Diago (3) 2012	1.192	(0.647,	2.195)
Peñarrocha−Oltra (1) 2012	0.828	(0.159,	4.315)
Peñarrocha−Oltra (2) 2013	9.895	(0.525,	186.347)
Piek 2013	1.998	(0.622,	6.415)
Pieri 2009	1.448	(0.089,	23.625)
Polizzi (1) 2000	1.011	(0.279,	3.670)
Polizzi (2) 2015	1.364	(0.187,	9.931)
Pozzi (1) 2014	0.418	(0.008,	21.349)
Pozzi (2) 2015	7.581	(0.356,	161.492)
Pozzi (3) 2016	3.677	(0.147,	91.741)
Prati (1) 2020	0.837	(0.039,	17.992)
Prati (2) 2020	3.061	(0.058,	160.412)
Raes (1) 2012	1.128	(0.069,	18.534)
Raes (2) 2013	6.290	(0.242,	163.416)
Raes (3) 2016	1.128	(0.069,	18.534)
Raes (4) 2018	1.476	(0.056,	38.837)
Ribeiro 2008	5.874	(0.294,	117.464)
Rodrigo 2012	1.000	(0.019,	51.844)
Romanos (1) 2012	0.462	(0.023,	9.249)
Romanos (2) 2014	6.281	(0.987,	39.970)
Romanos (3) 2014	0.488	(0.056,	4.232)
Saridakis 2018	1.459	(0.475,	4.479)
Sato 2014	3.585	(0.069,	186.278)
Schiegnitz 2016	1.516	(0.283,	8.119)
Schlee 2014	0.495	(0.027,	9.072)
Schoenbaum 2021	1.250	(0.612,	2.551)
Sennerby 2008	1.106	(0.122,	10.064)
Siciliano 2009	1.000	(0.019,	53.659)
Siebers 2010	12.074	(1.321,	110.374)
Swart 2014	0.344	(0.006,	18.464)
Tallarico (1) 2016	1.510	(0.060,	37.748)
Tallarico (2) 2017	1.000	(0.018,	54.465)
Testori 2014	2.508	(0.731,	8.609)
Thome (1) 2020	0.571	(0.036,	9.197)
Thome (2) 2021	0.606	(0.025,	14.969)
Tonetti 2017	3.000	(0.120,	75.191)
Urdaneta 2012	1.997	(0.528,	7.551)
Van Kesteren 2010	3.240	(0.120,	87.125)
Vandeweghe 2012	1.045	(0.104,	10.558)
Walton 2016	4.695	(0.222,	99.235)
Watzek 1995	0.182	(0.016,	2.074)
Wolfinger 2003	0.492	(0.080,	3.036)
Zafiropoulos 2010	3.031	(0.641,	14.334)
Zumstein 2016	5.545	(0.237,	129.833)
**Overall (I^2=0 % , P=0.532)**	1.349	(1.204,	1.512)

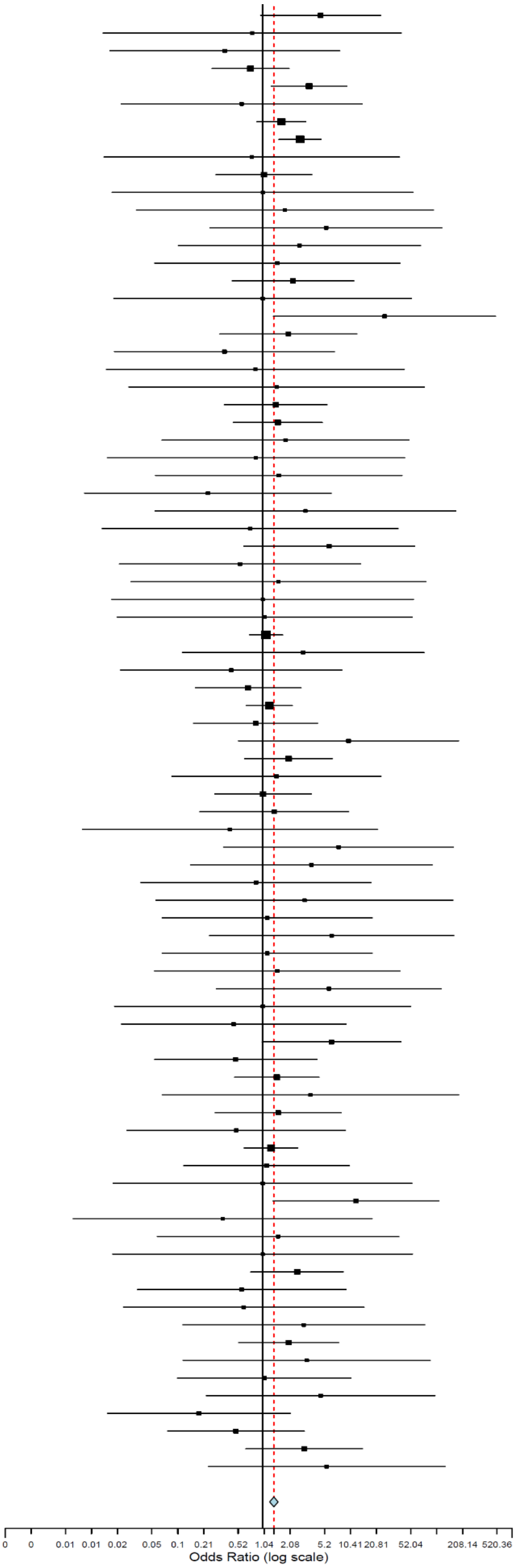

Figure S1. Forest plot for the event 'implant failure', global results.

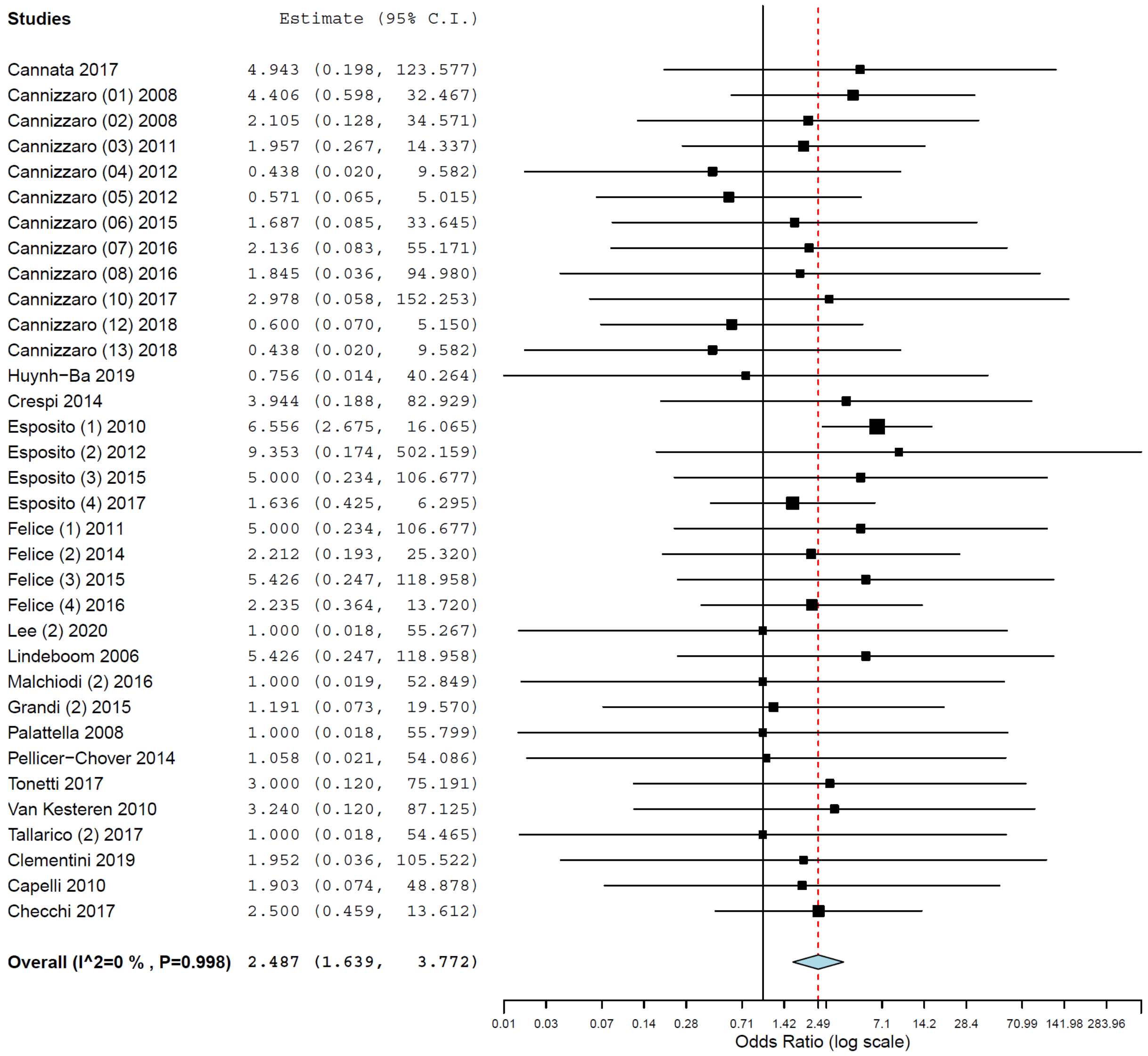

Figure S2. Forest plot for the event 'implant failure', when only results from RCT studies were pooled together.

Studies	Estimate (95% C.I.)
Agliardi 2014	0.658 (0.031, 13.970)
Agnini 2014	0.178 (0.009, 3.351)
Aguirre−Zorzano 2011	1.216 (0.048, 30.989)
Alves 2010	0.108 (0.005, 2.284)
Arora 2018	1.000 (0.019, 53.659)
Atieh (1) 2013	2.500 (0.361, 17.315)
Barbier 2012	1.000 (0.020, 51.232)
Bogaerde 2010	10.355 (0.401, 267.097)
Cannizzaro (09) 2016	5.320 (0.322, 87.884)
Cannizzaro (11) 2017	0.721 (0.033, 15.588)
Cooper 2014	3.288 (0.332, 32.609)
Cosyn (2) 2013	1.100 (0.202, 5.997)
Cucchi 2019	1.806 (0.386, 8.445)
Dayarpanah 2005	3.646 (0.370, 35.892)
De Bruyn (1) 2002	96.000 (11.815, 780.044)
De Bruyn (2) 2013	3.288 (0.332, 32.609)
Degidi (2) 2007	5.647 (0.496, 64.280)
Degidi (4) 2007	2.322 (0.046, 118.399)
Degidi (6) 2015	2.101 (0.514, 8.595)
Deng 2010	16.579 (0.862, 319.019)
Di Alberti 2012	1.784 (0.034, 92.650)
Gehrke 2015	1.000 (0.020, 51.218)
Grandi (1) 2013	2.087 (0.177, 24.615)
Horwitz 2007	4.688 (0.948, 23.166)
Irinakis 2009	0.365 (0.017, 7.810)
Krennmair 2014	0.748 (0.015, 38.468)
Luongo 2014	1.476 (0.056, 38.837)
Maló (2) 2003	1.984 (0.321, 12.276)
Meizi 2014	1.413 (0.359, 5.565)
Mertens 2011	1.844 (0.068, 49.695)
Norton (1) 2004	0.232 (0.009, 6.224)
Norton (2) 2017	3.133 (0.057, 171.963)
Peñarrocha−Oltra (2) 2013	9.895 (0.525, 186.347)
Piek 2013	1.998 (0.622, 6.415)
Pieri 2009	1.448 (0.089, 23.625)
Polizzi (1) 2000	1.011 (0.279, 3.670)
Pozzi (2) 2015	7.581 (0.356, 161.492)
Prati (1) 2020	0.837 (0.039, 17.992)
Prati (2) 2020	3.061 (0.058, 160.412)
Raes (1) 2012	1.128 (0.069, 18.534)
Raes (2) 2013	6.290 (0.242, 163.416)
Raes (3) 2016	1.128 (0.069, 18.534)
Raes (4) 2018	1.476 (0.056, 38.837)
Rodrigo 2012	1.000 (0.019, 51.844)
Sato 2014	3.585 (0.069, 186.278)
Schlee 2014	0.495 (0.027, 9.072)
Siciliano 2009	1.000 (0.019, 53.659)
Siebers 2010	12.074 (1.321, 110.374)
Swart 2014	0.344 (0.006, 18.464)
Walton 2016	4.695 (0.222, 99.235)
**Overall (I^2=0 % , P=0.836)**	2.070 (1.471, 2.912)

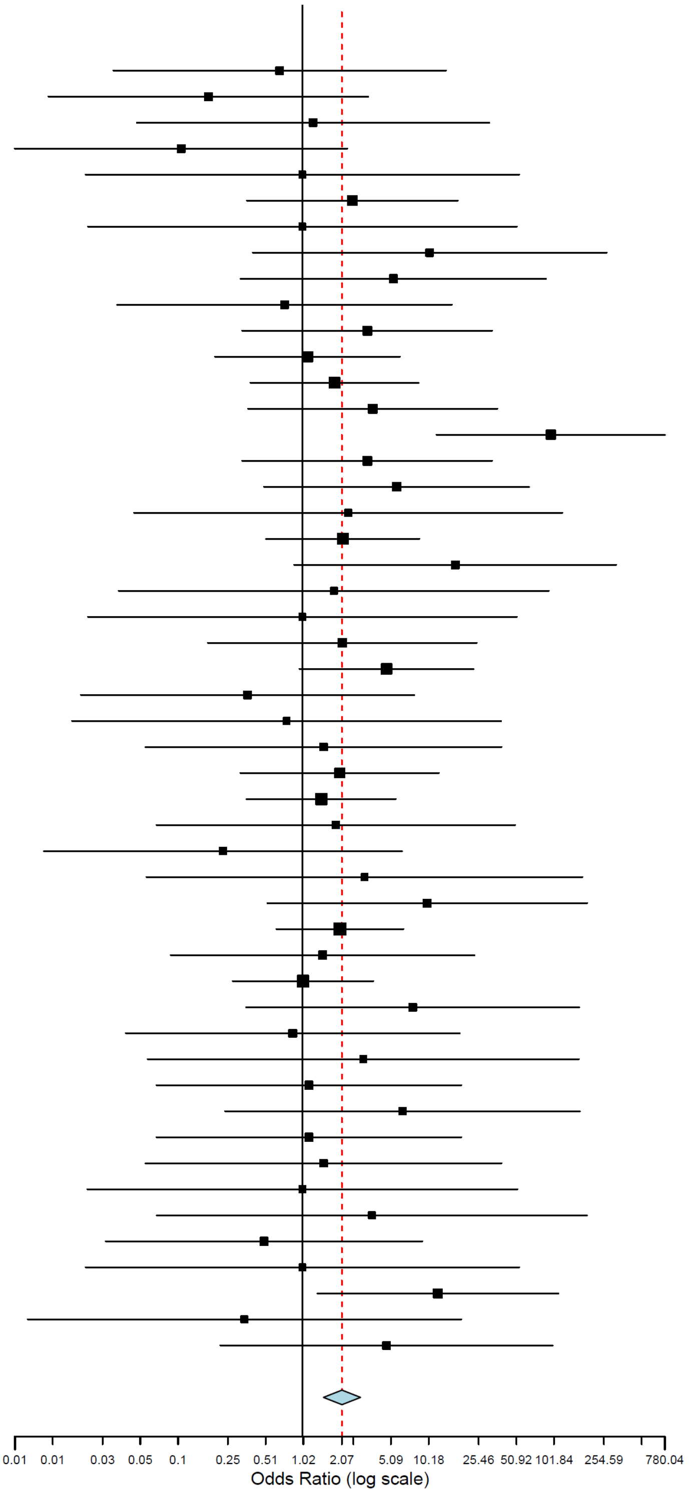

Figure S3. Forest plot for the event 'implant failure', when only results from prospective non-RCT studies were pooled together.

Table S1. Detailed data of the included studies.

Study	Year	Study design	Country / Setting	Patients (male/female) (n)	Patients' Age Range (mean) (years)	Prosthetic loading	Implants location	Implant used	Were there smokers in the group of patients? [a]
**Agliardi**	2014	CCT (unicenter)	Italy / Private practice	32 (15/17)	44-68 (58)	Immediate	Mx	MK IV and NobelSpeedy Groovy (Nobel Biocare, Göteborg, Sweden)	11 light smokers
**Agnini**	2014	CCT (unicenter)	Italy / University	30 (10/20)	NM (64.4)	Immediate	Mx, Md	Tapered Screw-Vent and Spline implants (Zimmer Dental Inc., Carlsbad, USA)	7 light and heavy smokers
**Aguirre-Zorzano**	2011	CCT (unicenter)	Spain / University	57 (19/37)	26-78 (48.5)	Immediate	Mx, Md	Osseospeed (Astra Tech Dental, Mölndal, Sweden)	NM
**Aires**	2002	RA (unicenter)	USA / NM	7 (NM)	NM	Immediate	Mx, Md	NM	NM
**Alves**	2010	CCT (unicenter)	Portugal / Private practice	23 (12/11)	NM	Immediate, delayed	Mx, Md	Several (Straumann, Nobel Biocare, Biomet 3i, Lifecore)	4 smokers
**Amato**	2020	RA (multicenter)	Italy / Private practice	55 (17/38)	30-86 (63)	Immediate	Mx, Md	T3 (Zimmer Biomet, Warsaw, USA)	16 smokers
**Andersson**	2015	RA (multicenter)	Sweden / Private practice	55 (27/28)	NM	Early (1-3 d)	Mx, Md	Bimodal and Proactive (Neoss Ltd, Harrogate, UK)	NM
**Annibali**	2011	RA (unicenter)	Italy / University	47 (19/18)	20-62 (40)	Mean 4 mo	Mx, Md	Nobel Replace TiUnite (Nobel Biocare, Göteborg, Sweden), Pilot ZirTi (Sweden & Martina, Due Carrare, Italy)	Only light smokers, but exact number not informed
**Arlin**	2002	RA (unicenter)	Canada / Private practice	961 (NM)	15-70 (51)	NM	Mx, Md	Several (Sulzer Dental, Nobel Biocare, Implant Innovations, Lifecore Biomedical, Straumann)	Yes, but exact number not informed
**Arora**	2018	PS (unicenter)	Australia / Private practice	30 (13/17)	26-77 (52)	Delayed (3-4 mo)	Mx	NM (Straumann, Basel, Switzerland)	1 light smoker
**Artzi**	2010	RA (unicenter)	Israel / University	54 (23/31)	34-81 (57.5)	Immediate	Mx, Md	DFI, ITO, and SPI (Alpha-Bio Tec, Petach Tikva, Israel)	21 heavy smokers
**Atieh**	2013	CCT (unicenter)	New Zealand / University	24 (9/15)	NM (52)	Immediate	Md	NM (Max Southern Implants, Irene, South Africa)	No
**Bae**	2011	RA (multicenter)	South Korea / Private practice + University	92 (41/51)	21-71 (42)	Delayed (3-7 mo)	Mx, Md	Seven (MIS Implants Technologies Ltd., Tel Aviv, Israel)	NM
**Barbier**	2012	CCT (unicenter)	Belgium / University	20 (11/9)	46-87 (61)	Immediate	Mx	Osseospeed (AstraTech AB, Mölndal, Sweden)	4 smokers
**Bettach**	2015	RA (unicenter)	France / Private practice	149 (79/70)	20-80 (51.8)	Immediate, delayed	Mx, Md	ID ALL (Implant Diffusion International)	Yes, but exact number not informed
**Bogaerde**	2010	CCT (multicenter)	Italy / Private practice	21 (9/12)	32-79 (60)	Within 7 days	Mx, Md	Bimodal (Neoss Ltd., Harrogate, UK)	Yes, but exact number not informed
**Cakarer**	2014	RA (unicenter)	Turkey / University	274 (NM)	19-84 (50)	Delayed (2-3 mo)	Mx, Md	Several (Astra Tech, Straumann, SwissPlus)	Yes, but exact number not informed
**Callan**	2000	RA (multicenter)	USA / Private practice	663 (269/394)	16-90 (55.3)	Delayed (3-6 mo)	Mx, Md	NM	175 smokers

**Cannata**	2017	RCT (multi-center)	Italy, Lebanon / Private practice	90 (43/47)	23-79 (51)	Delayed (3 mo)	Mx, Md	JDEvolution and JDIcon (J Dental Care, Modena, Italy)	17 light smokers<br>10 heavy smokers
**Cannizzaro**	2008	RCT (unicen-ter)	Italy / Private practice	30 (15/15)	42-75 (59)	Immedi-ate, early (2 mo)	Mx	Tapered SwissPlus (Zimmer Dental, Carlsbad, USA)	8 light smokers<br>7 heavy smokers
**Cannizzaro**	2008	RCT (unicen-ter)	Italy / Private practice	60 (25/35)	36-80 (61)	Immedi-ate, early (6 wk)	Md	Tapered SwissPlus (Zimmer Dental, Carlsbad, USA)	9 light smokers<br>21 heavy smokers
**Cannizzaro**	2011	RCT (unicen-ter)	Italy / Private practice	40 (20/20)	22-65 (44.5)	Immediate	Mx, Md	Tapered SwissPlus (Zimmer Dental, Carlsbad, USA)	12 light smokers<br>8 heavy smokers
**Cannizzaro**	2012	RCT (unicenter)	Italy / Private practice	30 (15/15)	18-57 (35)	Immedi-ate, de-layed (6 wk)	Mx, Md	NanoTite (Biomet 3i, Palm Beach, USA)	9 light smokers<br>3 heavy smokers
**Cannizzaro**	2012	RCT (unicen-ter)	Italy / Private practice	50 (24/26)	18-71 (38.8)	Immediate	Mx, Md	NanoTite (Biomet 3i, Palm Beach Gardens, USA)	12 light smokers<br>4 heavy smokers
**Cannizzaro**	2015	RCT (unicen-ter)	Italy / Private practice	60 (31/29)	38-80 (59)	Immediate	Mx, Md	Supershort and NT NanoTite (Biomet 3i, Palm Beach Gardens, USA)	7 light smokers<br>4 heavy smokers
**Cannizzaro**	2016	RCT (unicen-ter)	Italy / Private practice	50 (25/25)	19-62 (40)	Immediate	Mx, Md	Syra and Syra SL (Sweden & Martina, Due Carrare, Italy)	11 light smokers<br>6 heavy smokers
**Cannizzaro**	2016	RCT (unicen-ter)	Italy / Private practice	40 (21/19)	33-78 (56)	Immediate	Mx, Md	Prama PF tapered (Sweden & Mar-tina, Due Carrare, Italy)	12 light smokers<br>4 heavy smokers
**Cannizzaro**	2016	PS (unicen-ter)	Italy / Private practice	80 (40/40)	29-85 (53.2)	Immediate	Md	Tapered NT NanoTite (Biomet 3i, Palm Beach Gardens, USA)	18 light smokers<br>16 heavy smokers
**Cannizzaro**	2017	RCT (multi-center)	Italy / Private practice	60 (29/31)	37-83 (59)	Immediate	Md	NT Full Osseotite (Zimmer Biomet, Palm Beach Gardens, USA), ExFeel (MegaGen Implant, Gyeongbuk, South Korea)	11 light smokers<br>5 heavy smokers
**Cannizzaro**	2017	PS (unicen-ter)	Italy / Private practice	40 (18/22)	NM (59)	Immediate	Mx, Md	Syra and Syra SL (Sweden & Martina, Due Carrare, Italy)	13 light smokers<br>4 heavy smokers
**Cannizzaro**	2018	RCT (multi-center)	Italy / Private practice	48 (28/20)	36-78 (58)	Immediate	Mx, Md	Syra and Syra SL (Sweden & Martina, Due Carrare, Italy)	13 light smokers<br>4 heavy smokers
**Cannizzaro**	2018	RCT (unicenter)	Italy / Private practice	30 (15/15)	18-57 (35)	Immedi-ate, de-layed (6 wk)	Mx, Md	NanoTite (Biomet 3i, Palm Beach Gardens, USA)	9 light smokers<br>3 heavy smokers
**Capelli**	2010	RCT (multi-center)	Italy / Private practice	52 (23/29)	27-74 (51)	Immedi-ate, early (2 mo)	Mx, Md	Full Osseotite Tapered (Zimmer Biomet, Palm Beach Gardens, USA)	12 light smokers<br>1 heavy smokers
**Carr**	2019	RA (unicen-ter)	USA / Non-profit organiza-tion	2798 (NM)	>18	Immedi-ate, de-layed	Mx, Md	NM	Yes, but exact number not informed
**Cavallaro**	2011	RA (unicenter)	USA / Private practice	75 (33/42)	28-84 (61.8)	Delayed (6-8 wk)	Mx, Md	NM (Implant Direct LLC, Calabasas, USA)	18 smokers
**Cercadillo-Ibarguren**	2017	RA (unicen-ter)	Spain / Private practice	88 (40/48)	40-84 (62.4)	Immedi-ate, de-layed	Mx, Md	Replace Select Tapered TiUnite (No-bel Biocare, Göteborg, Sweden)	2 light smokers<br>26 heavy smokers
**Chaushu**	2001	RA (unicenter)	Israel / Private practice	26 (6/20)	18-70 (44)	Immediate	Mx, Md	Steri-Oss (Steri-Oss Company, Yorba Linda, USA), NM (Alpha Bio, Petah-Tikva, Israel)	No

**Checchi**	2017	RCT (multi-center)	Italy / Private practice + University	100 (51/49)	NM (53)	Delayed (4 mo)	Mx, Md	Rescue (MegaGen Implant, Gyeongbuk, South Korea)	17 light smokers<br>7 heavy smokers
**Ciabattoni**	2017	RA (multi-center)	Italy / Private practice	32 (9/23)	44-73 (59.5)	Immediate	Mx, Md	Mk III TiUnite, NobelActive, Speedy Groovy, Nobel Replace (Nobel Biocare, Göteborg, Sweden)	Light smokers, but the exact number was not informed
**Clementini**	2019	RCT (multi-center)	Italy / University	30 (14/16)	NM (52)	Not loaded	Mx, Md	TTi WINSIX (Biosafin, Ancona, Italy)	10 light smokers
**Cooper**	2014	CCT (multi-center)	USA, Spain, Germany, Belgium / University	113 (47/66)	NM (42-45)	Immediate	Mx	Osseospeed (AstraTech AB, Mölndal, Sweden)	No
**Correia**	2017	RA (unicenter)	Portugal / Private practice	202 (70/132)	23-73 (50)	Immediate, delayed	Mx, Md	Several (Straumann, Nobel Biocare, Biomet 3i, Neodent, Klockner, EuroTeknika)	15 light smokers<br>29 heavy smokers
**Cosyn**	2012	RA (unicenter)	Belgium / University	461 (216/245)	18-90 (51)	Immediate, within 3 mo, delayed	Mx, Md	Several (Nobel Biocare, Straumann, Dentsply Friadent, Astra Tech, Biomet 3i)	Yes, but exact number not informed
**Cosyn**	2013	CCT (multicenter)	Belgium / University	104 (43/61)	22-80 (51)	Immediate, delayed (3-6 mo)	Mx	TiUnite NobelReplace (Nobel Biocare, Göteborg, Sweden)	18 smokers
**Covani**	2012	RA (unicenter)	Italy / Private practice	19 (6/13)	39-72 (60)	Immediate	Mx, Md	Ossean, (Intra-Lock International, Inc, Boca Raton, USA)	Light smokers, but exact number not informed
**Crespi**	2014	RCT (unicenter)	Italy / University	28 (13/15)	46-77 (59.3)	Immediate	Mx, Md	Outlink (Sweden & Martina, Due Carrare, Italy)	Light smokers, but the exact number was not informed
**Cucchi**	2019	PS (NM)	Italy / NM	20 (NM)	NM (67)	Early (3-5 d)	Mx, Md	NM	28% of the implants in smokers
**Davarpanah**	2005	CCT (unicenter)	France / Private practice	92 (36/56)	NM (59.8)	Delayed (3-6 mo)	Mx, Md	Osseotite NT (Biomet 3i, Palm Beach Gardens, USA)	Yes, but exact number not informed
**De Bruyn**	2002	CCT (unicenter)	Belgium / University	36 (18/18)	63-81 (NM)	Within 8 days	Md	Brånemark (Nobel Biocare AB, Göteborg, Sweden)	Yes, but exact number not informed
**De Bruyn**	2013	CCT (multicenter)	Belgium, Spain, USA, Germany / University	113 (47/66)	NM (42-45)	Immediate	Mx	Osseospeed (Astra Tech AB, Mölndal, Sweden)	No
**Degasperi**	2014	RA (NM)	Italy / Private practice	49 (20/49)	29-79 (50.9)	Delayed (3-4 mo)	Mx, Md	Neoss Proactive (Neoss Ltd, Harrogate, UK)	NM
**Degidi**	2006	RA (unicenter)	Italy / University	111 (46/65)	15-83 (40)	Immediate	Mx, Md	Several	19 light and heavy smokers
**Degidi**	2007	CCT (unicenter)	Italy / University	NM	15-83 (55)	Immediate	Mx, Md	Several	Yes, but exact number not informed
**Degidi**	2007	RA (unicenter)	Italy / University	133 (NM)	41-80 (56)	Immediate	Mx, Md	Several	Yes, but exact number not informed
**Degidi**	2007	PS (unicenter)	Italy / University	50 (22/28)	42-77 (57.5)	Immediate	Md	Brånemark (Nobel Biocare, Göteborg, Sweden), Frialit, XiVE, IMZ, and Frialoc (Dentsply Friadent), Maestro (BioHorizons), Restore (Lifecore)	Light and heavy smokers, but the exact number was not informed

**Degidi**	2012	RA (unicenter)	Italy / University	1045 (NM)	18-93 (NM)	NM	Mx, Md	XiVe (Dentsply Friadent, Mannheim, Germany)	Yes, but exact number not informed
**Degidi**	2015	PS (unicenter)	Italy / Private practice	114 (NM)	NM (53.1)	Immediate	Mx, Md	XiVE (Dentsply, Mannheim, Germany)	34 smokers
**Deng**	2010	CCT (unicenter)	China / University	12 (4/8)	40-75 (62)	Immediate	Mx, Md	TiUnite, Brånemark System Mk III, NobelSpeedy (Nobel Biocare, Göteborg, Sweden)	9 smokers
**Dhanrajani**	2005	RA (unicenter)	Saudi Arabia / Private practice	101 (30/71)	17-69 (NM)	Immediate, delayed	Mx, Md	Brånemark (Nobel Biocare AB, Göteborg, Sweden), Osseotite NT (Biomet 3i, Palm Beach Gardens, USA), Omniloc (Calcitek Sulzer, Carlsbad, USA), Steri-Oss, Replace (Nobel Biocare AB, Göteborg, Sweden)	17 light smokers<br>7 heavy smokers
**Di Alberti**	2012	CCT (unicenter)	Italy / Private practice	70 (33/37)	16-71 (45)	Immediate	Mx	Seven (MIS)	NM
**Esposito**	2010	RCT (multicenter)	Italy / Private practice	506 (236/270)	18-86 (48)	Not loaded	Mx, Md	Several	115 light smokers<br>54 heavy smokers
**Esposito**	2012	RCT (multicenter)	Italy / Private practice	60 (31/29)	25-81 (53)	Early (within 2 wk)	Mx	Xpeed (MegaGen Implant, Gyeongbuk, South Korea)	8 light smokers<br>3 heavy smokers
**Esposito**	2015	RCT (multicenter)	Italy / Private practice	106 (46/60)	28-72 (49)	Immediate	Mx	EZ Plus (MegaGen Implant, Gyeongbuk, South Korea)	15 light smokers<br>7 heavy smokers
**Esposito**	2017	RCT (multicenter)	Italy / Private practice	210 (107/103)	29-79 (54)	Delayed (4 mo)	Mx, Md	NobelActive (Nobel Biocare, Göteborg, Sweden)	59 light smokers<br>16 heavy smokers
**Evian**	2004	RA (unicenter)	USA / University	149 (NM)	NM	Delayed (4-6 mo)	Mx, Md	NM (Paragon, Zimmer Dental, Carlsbad, USA)	NM
**Feher**	2020	RA (unicenter)	Austria / University	1132 (505/627)	NM (50.6)	Not loaded	Mx, Md	NM	157 light smokers<br>60 heavy smokers
**Felice**	2011	RCT (multicenter)	Italy / Private practice	106 (46/60)	28-72 (49)	Immediate, delayed (4 mo)	Mx	EZ Plus (MegaGen, Gyeongbuk, South Korea)	15 light smokers<br>7 heavy smokers
**Felice**	2014	RCT (multicenter)	Italy / Private practice	64 (35/29)	19-80 (52)	Immediate, early, delayed	Mx, Md	Way Milano and Kentron (Geass srl, Pozzuolo Del Friuli, Udine, Italy)	11 light smokers<br>6 heavy smokers
**Felice**	2015	RCT (multicenter)	Italy / Private practice	50 (25/25)	32-72 (52)	Immediate, delayed (4 mo)	Mx	XiVE S plus (Dentsply Friadent, Mannheim, Germany)	17 light smokers<br>3 heavy smokers
**Felice**	2016	RCT (multicenter)	Italy / Private practice + University	150 (66/84)	20-86 (54)	Delayed (3 mo)	Mx, Md	TwinKon Universal SA2 (Global D, Lyon, France)	33 light smokers<br>7 heavy smokers
**Gehrke**	2015	CCT (unicenter)	Brazil / NM	77 (24/53)	26-65 (NM)	Not loaded	Mx, Md	NM (Implacil De Bortoli, São Paulo, Brazil)	No
**Gelb**	2013	RA (multicenter)	USA / Private practice	52 (21/31)	35-82 (64.1)	Delayed (4-12 mo)	Mx, Md	TiUnite (Nobel Biocare, Göteborg, Sweden)	3 smokers
**Gillot**	2011	RA (multicenter)	France / Private practice	105 (53/52)	NM (58.7)	Immediate	Md	TiUnite (Nobel Biocare, Göteborg, Sweden)	Smokers (<15 cig/day) included, but the exact number was not informed

**Gillot**	2012	RA (multi-center)	France / Private practice	113 (56/57)	37-93 (60.4)	Immediate	Mx	TiUnite MkIII, MkIV, Speedy, NobelActive (Nobel Biocare, Göteborg, Sweden)	Smokers (<15 cig/day) included, but the exact number was not informed
**Giordano**	2017	RA (unicenter)	Italy / Private practice	104 (43/61)	37-87 (61.2)	Immediate	Mx, Md	SPI-Contact (Thommen Medical, Grenchen, Switzerland)	20 light smokers<br>24 heavy smokers
**Gomez-Roman**	1997	RA (unicenter)	Germany / University	376 (199/177)	15-88 (38.8)	Delayed (3-8 mo)	Mx, Md	Frialit-2 (Dentsply Friadent, Mannheim, Germany)	NM
**Grandi**	2013	CCT (multi-center)	Italy / Private practice	50 (21/29)	31-73 (56)	Immediate	Mx	JD Evolution (J DentalCare, Modena, Italy)	19 light and heavy smokers
**Grandi**	2015	RCT (multi-center)	Italy / Private practice	105 (50/55)	21-75 (47)	Immediate, early, delayed	Mx, Md	JD Evolution (J DentalCare, Modena, Italy)	19 light smokers<br>6 heavy smokers
**Guarnieri**	2020	RA (multi-center)	Italy / Private practice	274 (146/128)	45-75 (48.1)	Immediate, delayed	Mx, Md	BioHorizons TLX and TRX (BioHorizons)	124 smokers
**Haas**	1996	RA (unicenter)	Austria / University	607 (246/361)	21-86 (51.5)	Delayed (3-6)	Mx, Md	IMZ (Friatec, Friedrichseld, Germany)	NM
**Horwitz**	2007	CCT (unicenter)	Israel / Private practice	19 (2/17)	34-79 (NM)	Delayed (6 mo)	Mx, Md	NM (MIS Implant Technologies, Shlomi, Israel)	NM
**Huynh-Ba**	2019	RCT (unicenter)	USA / University	35 (18/17)	27-74 (52)	Delayed (4 mo)	Mx, Md	Bone Level SLActive (Straumann, Basel, Switzerland)	Light smokers, but exact number not informed
**Irinakis**	2009	CCT (multicenter)	Canada / Private practice	67 (NM)	NM	Immediate, delayed	Mx, Md	NobelActive (Nobel Biocare, Göteborg, Sweden)	NM
**Jaffin**	2004	RA (unicenter)	USA / University	34 (19/15)	43-82 (60)	Immediate	Mx	SLA (Straumann, Waldenburg, Switzerland)	Yes, but exact number not informed
**Ji**	2012	RA (unicenter)	USA / University	45 (18/27)	25-88 (61.5)	Immediate	Mx, Md	TiUnite (Nobel Biocare, Göteborg, Sweden), NM (Zimmer Dental, Carlsbad, USA), XiVe (Dentsply Friadent, Mannheim, Germany)	8 smokers
**Kim**	2017	RA (unicenter)	South Korea / University	116 (55/61)	17-76 (50.9)	Delayed (10-22 wk)	Mx, Md	Oneplant (Warantec, Seoul, Republic of Korea) IS-II (Neobiotech, Seoul, Republic of Korea)	NM
**Kohen**	2016	RA (unicenter)	Israel / Private practice	343 (NM)	> 18	Immediate, early, delayed	Mx, Md	Several (SPI; DFI; Arrow, Alph-Bio-Tec; screw-vent TSV, Zimmer Dental; Maestro, BioHorizons HPI Inc)	NM
**Kourtis**	2004	RA (multicenter)	Greece / Private practice + University	405 (171/234)	18-83 (54.3)	Immediate, delayed	Mx, Md	Several (IMZ, Frialit-2, Free-Hex, Frialoc, Friadent Co., Mannheim, Germany)	Yes, but exact number not informed
**Krennmair**	2014	PS (unicenter)	Austria / University	24 (14/10)	38-84 (61.5)	Immediate	Md	Screw-Line Promote plus (Camlog, Wimsheim, Germany)	Light smokers, but exact number not informed
**Lee**	2018	RA (unicenter)	Taiwan / Private practice	161 (NM)	23-90 (53)	Immediate, delayed	Mx, Md	IDEOSS (IDEOSS Biotech, Taipei, Taiwan)	Yes, but the exact number was not reported
**Lee**	2020	RCT (unicenter)	Switzerland / University	20 (12/8)	≥ 22	Delayed (8 wk)	Mx, Md	BLT (Straumann, Basel, Switzerland)	NM
**Leonida**	2012	RA (unicenter)	Italy / University	9 (1/8)	45-68 (NM)	Immediate	Md	Way (Geass, Pozzuolo del Friuli, Italy)	No

**Lindeboom**	2006	RCT (unicenter)	Netherlands / University	50 (25/25)	19-69 (39.7)	Delayed (6 mo)	Mx	Frialit-2 Synchro (Dentsply Friadent, Mannheim, Germany)	No
**Locante**	2004	RA (unicenter)	USA / Private practice	86 (35/51)	12-81 (NM)	Immediate	Mx	Stabledent (Crystal Medical Technology, Pelham, USA)	9 smokers
**Luongo**	2014	CCT (multicenter)	Italy / Private practice	46 (23/23)	18-73 (44.5)	Immediate	Mx, Md	AnyRidge (MegaGen, Gyeongbuk, South Korea)	17 smokers
**Malchiodi**	2011	RA (unicenter)	Italy / University	81 (NM)	NM	Immediate	Mx	Osseotite Certain (Biomet 3i, Palm Beach Gardens, USA), FBR Pitt-Easy (Oraltronics, Bremen, Germany)	Yes, but exact number not informed
**Malchiodi**	2016	RCT (unicenter)	Italy / University	40 (24/16)	35-75 (52)	Delayed (3 mo)	Mx, Md	SybronPRO XR (Sybron Implant Solutions)	10 light and heavy smokers
**Maló**	2000	RA (unicenter)	Portugal / Private practice	49 (18/31)	16-64 (42)	Immediate	Mx, Md	Brånemark MkII (Nobel Biocare AB, Göteborg, Sweden)	Light smokers, but exact number not informed
**Maló**	2003	CCT (multicenter)	Portugal, Sweden, Italy / Private practice + Public service	76 (41/35)	18-81 (41)	Immediate	Mx, Md	Brånemark (Nobel Biocare AB, Göteborg, Sweden)	24 heavy smokers
**Maló**	2003	RA (unicenter)	Portugal / Private practice	44 (15/29)	30-79 (59)	Immediate	Md	Brånemark MkII and MkIII (Nobel Biocare AB, Göteborg, Sweden)	NM
**Mangano**	2013	RA (unicenter)	Italy / Private practice	40 (26/14)	18-62 (45)	Immediate	Mx	NM (Sistema Implantare Leone, Sesto Fiorentino, Italy)	Patients smoking less than 15 cig./day
**Mangano**	2017	RA (multicenter)	Brazil, Italy / Private practice	103 (43/60)	24-65 (41.4)	Immediate	Mx	Tixos (Leader Implants, Milan, Italy)	No
**Meizi**	2014	CCT (unicenter)	Israel / NM	155 (40/95)	20-70 (47.5)	Immediate, delayed (3-6 mo)	Mx, Md	Saturn (Cortex Dental, Shlomi, Israel)	12 heavy smokers
**Mensdorff-Pouilly**	1994	RA (unicenter)	Austria / University	31 (12/19)	NM	Delayed (3-6 mo)	Mx, Md	IMZ (Friatec, Mannheim, Germany), Brånemark (Nobel Biocare, Göteborg, Sweden)	NM
**Mertens**	2011	CCT (unicenter)	Germany / Univeristy	17 (5/12)	40-83 (61)	Immediate, delayed (mean 9.5 wk)	Mx, Md	Osseospeed (AstraTech AB, Mölndal, Sweden)	2 smokers
**Moraes**	2013	RA (multicenter)	Brazil / University, private practice	27 (9/18)	25-80 (56)	Delayed (4-6 mo)	Mx, Md	Way Syntegra (Geass, Udine, Italy)	NM
**Noelken**	2014	RA (unicenter)	Germany / Private practice	20 (10/10)	31-69 (NM)	Immediate	Mx, Md	NobelPerfect (Nobel Biocare, Göteborg, Sweden)	5 smokers
**Norton**	2004	CCT (unicenter)	UK / Private practice	25 (10/15)	27-72 (48.2)	Immediate	Mx	TiO-Blast (AstraTech ST, Mölndal, Sweden)	Yes, but exact number not informed
**Norton**	2017	PS (unicenter)	United Kingdom / Private practice	22 (10/12)	22-79 (NM)	Immediate	Mx, Md	Astra Tech AV (Dentsply, Mölndal, Sweden)	1 light smoker
**Oliva**	2012	RA (unicenter)	Spain / Private practice	17 (11/6)	39-79 (52.9)	Delayed (4 mo)	Mx, Md	Straumann (Straumann, Basel, Switzerland), Osstem (Osstem, Seoul, South Korea)	8 smokers

**Ormianer**	2008	RA (multicenter)	Israel / Private practice	60 (26/34)	18-78 (53)	Immediate, delayed	Mx, Md	Tapered Screw-Vent MTX (Zimmer Dental, Inc, Carlsbad, USA)	2 smokers
**Ormianer**	2012	RA (unicenter)	Israel / Private practice	46 (19/27)	NM (50)	Immediate, delayed	Mx	Tapered Screw-Vent MTX (Zimmer Dental, Inc, Carlsbad, USA)	1 smoker
**Oxby**	2015	RA (unicenter)	Sweden / Private practice	39 (17/22)	> 20 (68)	Early (within 60 d)	Mx, Md	OsseoSpeed (Astra Tech, Dentsply Implants, Mölndal, Sweden)	1 extra-heavy smoker
**Palattella**	2008	RCT (unicenter)	Italy / University	16 (6/10)	21-49 (35)	Immediate	Mx	SLA (Straumann, Basel, Switzerland)	Only light smokers, but exact number not informed
**Pellicer-Chover**	2014	RCT (unicenter)	Spain / University	15 (9/6)	NM (63.7)	Delayed (10-12 wk)	Mx, Md	Kohno SP (Sweden & Martina, Due Carrare, Italy)	No
**Peñarrocha-Diago**	2008	RA (unicenter)	Spain / University	100 (45/55)	20-76 (47.5)	Delayed (6-8 wk)	Mx, Md	Defcon Avantblast, Impladent (Sentmenat, Barcelona, Spain)	10 light smokers 31 heavy smokers
**Peñarrocha-Diago**	2011	RA (unicenter)	Spain / University	30 (13/17)	36-68 (53.2)	Delayed (6-10 wk)	Mx, Md	Defcon Avantblast TSA, Impladent (Senmenat, Barcelona, Spain)	Light smokers, but exact number not informed
**Peñarrocha-Diago**	2012	RA (unicenter)	Spain / University	150 (73/77)	25-80 (55.4)	Delayed (2-3 mo)	Mx, Md	Defcon implants (Impladent SL, Barcelona, Spain)	NM
**Peñarrocha-Oltra**	2012	RA (unicenter)	Spain / University	70 (32/38)	34-75 (54)	Delayed (8-10 wk)	Mx	NM	27 light smokers
**Peñarrocha-Oltra**	2013	CCT (unicenter)	Spain / University	29 (13/16)	28-77 (55.4)	Immediate, delayed (2 mo)	Mx	Kohno SP (Sweden & Martina, Due Carrare, Italy)	No
**Perry**	2004	RA (multicenter)	USA / Private practice	442 (194/248)	17-92 (55)	Delayed (3-5 mo)	Mx, Md	Frialit-2 (Dentsply Friadent, Mannheim, Germany)	NM
**Pettersson**	2015	RA (unicenter)	Sweden / Private practice	88 (32/56)	NM (65)	Delayed (3-4 mo)	Mx, Md	Replace Select TiUnite (Nobel Biocare, Göteborg, Sweden)	Yes, but exact number not informed
**Piek**	2013	CCT (unicenter)	Israel / University	141 (58/83)	NM (57.6)	Immediate, delayed	Mx, Md	NM (Paltop Advanced Dental Solutions Ltd., Caesarea, Israel)	16 smokers
**Pieri**	2009	CCT (unicenter)	Italy / Private practice	23 (10/13)	51-72 (61.9)	Immediate	Mx, Md	NM (Keystone Dental, Burlington, USA)	Light smokers, but exact number not informed
**Polizzi**	2000	CCT (multicenter)	Many countries / Private practice+ University	143 (68/75)	NM (47, females) NM (40, males)	Delayed (3-6 mo)	Mx, Md	Brånemark (Nobel Biocare AB, Göteborg, Sweden)	NM
**Polizzi**	2015	RA (unicenter)	Italy / Private practice	27 (7/20)	34-71 (55.8)	Immediate	Mx	Nobel Speedy Groovy, Nobel Replace Select Tapered (Nobel Biocare, Göteborg, Sweden)	Yes, but exact number not informed
**Pozzi**	2014	RA (unicenter)	Italy / Private practice	73 (32/41)	19-76 (50.59)	Immediate, delayed	Mx, Md	MK III, MK IV, Nobel Replace Select Tapered (Nobel Biocare, Göteborg, Sweden)	NM
**Pozzi**	2015	PS (unicenter)	Italy / University	54 (22/32)	23-80 (56.2)	Immediate	Mx, Md	Nobel Replace Conical Connection (Nobel Biocare, Göteborg, Sweden)	3 light smokers
**Pozzi**	2016	RA (multicenter)	Italy / Private practice	64 (26/38)	21-80 (52.8)	Immediate	Mx, Md	Nobel Replace Conical Connection (Nobel Biocare, Göteborg, Sweden)	Only light smokers, but exact number not informed

**Prati**	2020	PS (multi-center)	Italy / University + Private practice	76 (34/42)	NM (55.6)	Delayed (3 mo)	Mx, Md	Premium SP (Sweden & Martina, Due Carrare, Italy)	8 heavy smokers
**Prati**	2020	PS (multi-center)	Italy / University + Private practice	56 (27/29)	NM (55)	Delayed (3 mo)	Mx, Md	Prama (Sweden & Martina, Due Carrare, Italy)	Only light smokers, but exact number not informed
**Raes**	2012	CCT (unicenter)	Belgium / University	96 (41/55)	18-72 (43)	Immediate	Mx	Osseospeed (AstraTech AB, Mölndal, Sweden)	No
**Raes**	2013	CCT (unicenter)	Belgium / University	48 (27/21)	NM	Immediate	Mx	Osseospeed (AstraTech AB, Mölndal, Sweden)	4 former smokers
**Raes**	2017	CCT (multi-center)	Belgium, USA, Spain / University	96 (41/55)	18-72 (43)	Immediate	Mx	OsseoSpeed (Astra Tech, Dentsply Implants, Mölndal, Sweden)	No
**Raes**	2018	PS (multi-center)	Italy, Belgium / Private practice + University	46 (23/23)	18-73 (44.5)	Immediate	Mx, Md	AnyRidge (MegaGen Implant, Gyeongbuk, South Korea)	17 smokers
**Ribeiro**	2008	RA (multicenter)	Brazil / University	64 (27/37)	23-71 (45.4)	Immediate	Mx	NM (Conexão, São Paulo, Brazil)	NM
**Rodrigo**	2012	CCT (unicenter)	Spain / Private practice	22 (8/14)	33-76 (59.3)	Immediate, delayed (2-4 mo)	Mx, Md	SLA (Straumann, Basel, Switzerland)	3 light smokers<br>3 heavy smokers<br>2 extra-heavy smokers
**Romanos**	2012	RA (unicenter)	Germany / Private practice	55 (20/35)	NM (63)	Immediate	Md	Ankylos (Friadent, Mannheim, Germany)	NM
**Romanos**	2014	RA (NM)	NM	27 (15/12)	NM (59)	Immediate	Mx, Md	Ankylos (Dentsply Implants, Mölndal, Sweden)	2 light smokers<br>6 extra-heavy smokers
**Romanos**	2014	RA (NM)	NM	26 (6/20)	NM (57)	Immediate	Mx	Ankylos (Dentsply Implants, Mölndal, Sweden)	NM
**Saridakis**	2018	RA (unicenter)	Germany / University	98 (NM)	> 18	Immediate, delayed	Mx, Md	Nobel Active (Nobel Biocare, Göteborg, Sweden)	No
**Sato**	2014	PS (multi-center)	Japan / University + Private practice	63 (25/38)	NM (54)	Immediate	Mx, Md	Nobel Direct (Nobel Biocare, Göteborg, Sweden)	14 smokers
**Schiegnitz**	2016	RA (unicenter)	Germany / University	90 (38/52)	22-87 (57.7)	NM	Mx, Md	Straumann TE (Straumann, Basel, Switzerland)	NM
**Schlee**	2014	PS (multi-center)	Netherlands, Germany, Italy, France, Spain / Private practice	105 (50/55)	22-77 (55.2)	NM	Mx, Md	Trabecular Meta Material (Zimmer TMT, Parsippany, USA)	17 light and heavy smokers
**Schoenbaum**	2021	RA (multi-center)	USA / Private practice	378 (181/197)	NM (60)	NM	Mx, Md	NM	15% of the implants in smokers and former smokers
**Sennerby**	2008	RA (multicenter)	Sweden, Italy, USA / University + Private practice	43 (20/23)	NM (50)	Immediate/early, delayed (6 wk-6 mo)	Mx, Md	TiUnite, NobelDirect (Nobel Biocare, Göteborg, Sweden)	NM
**Siciliano**	2009	CCT (unicenter)	Italy / University	30 (16/14)	NM (49)	Delayed (3 mo)	Mx, Md	Straumann TE (Straumann, Basel, Switzerland)	No

**Siebers**	2010	CCT (unicenter)	Germany / Private practice	76 (34/42)	22-85 (52)	Delayed (4-6 mo)	Mx, Md	Camlog Rootline and Screw Line (Camlog Biotechnologies, Basel, Switzerland), Osseotite (Biomet 3i, Palm Beach Gardens, USA), Restore RBM (Lifecore Biomedical, Chaska, USA)	15 smokers
**Swart**	2014	PS (unicenter)	South Africa / Private practice	8 (NM)	38-72 (60)	Immediate	Md	NM	Light and heavy smokers, but exact number not informed
**Tallarico**	2016	RA (NM)	NM	56 (25/31)	NM (66.2)	Immediate, delayed	Mx, Md	NobelReplace Conical Connection, NobelSpeedy Groovy, Brånemark MKIII, NobelReplace Tapered Groovy (Nobel Biocare, Göteborg, Sweden)	3 light smokers
**Tallarico**	2017	RCT (unicenter)	Italy / Private practice	24 (8/16)	37-67 (53)	Delayed (4 mo)	Mx, Md	Ultra-Wide (Osstem, Seoul, South Korea)	No
**Testori**	2014	RA (unicenter)	Italy / University	80 (38/42)	NM (59)	Immediate, delayed	Mx, Md	NM (Biomet 3i, Garden Beach, USA)	11 light smokers<br>15 heavy smokers
**Thome**	2020	RA (unicenter)	Brazil / Private practice	101 (48/53)	NM (59.2)	Immediate, delayed	Mx, Md	Helix Acqua GM (Neodent, Curitiba, Brazil)	NM
**Thome**	2021	RA (unicenter)	Brazil / University	67 (NM)	NM (60.6)	Immediate	Mx, Md	Helix Acqua GM (Neodent, Curitiba, Brazil)	NM
**Tonetti**	2017	RCT (multicenter)	Italy, China / Private practice + University	124 (40/84)	NM (50-55; 2 groups)	Delayed (12 wk)	Mx, Md	SPI Contact (Thommen Medical AG, Waldenburg, Switzerland)	18 smokers
**Urdaneta**	2012	RA (unicenter)	USA / Private practice	291 (147/144)	NM	NM	Mx, Md	Integra-CP (Bicon, Boston, USA)	Yes, but exact number not informed
**van Kesteren**	2010	RCT (unicenter)	USA / Private practice	24 (NM)	28-76 (NM)	Not loaded	Mx, Md	SLA (Straumann, Waldenburg, Switzerland)	Yes, but exact number not informed
**Vandewegh e**	2012	RA (multicenter)	Belgium, South Africa, Germany, UK/ University + Private practice	75 (31/44)	25-82 (58)	Immediate, delayed (3 mo)	Mx, Md	NM (Max Southern Implants, Irene, South Africa)	6 light smokers<br>4 heavy smokers
**Walton**	2016	PS (unicenter)	Australia / Private practice	184 (82/102)	15-79 (46.2)	Immediate, delayed (>3 mo)	Mx, Md	MK III, MK IV, Replace Select, NobelActive, Speedy Groovy TiUnite (Nobel Biocare, Göteborg, Sweden)	Yes, but exact number not informed
**Watzek**	1995	RA (unicenter)	Austria / University	20 (6/14)	NM	Delayed (3-6 mo)	Mx, Md	Brånemark (Nobel Biocare AB, Göteborg, Sweden), TPS (Friedrichsfeld AG, Mannheim, Germany)	NM
**Wolfinger**	2003	RA (unicenter)	USA / Private practice	10 (NM)	45-70 (55)	Immediate, delayed (3 mo)	Md	Brånemark (Nobel Biocare AB, Göteborg, Sweden)	NM
**Zafiropoulos**	2010	RA (unicenter)	Germany / Private practice	252 (127/114)	43-70 (49)	Immediate	Mx	Camlog root line (Altatec, Wimsheim, Germany), SLA (Straumann, Waldenburg, Switzerland)	86 smokers

**Zumstein**	2016	RA (unicenter)	Switzerland / Private practice	50 (22/28)	NM (58.6)	Immediate, delayed	Mx, Md	Proactive (Neoss Ltd, Harrogate, UK)	4 heavy smokers

NM—not mentioned; RA—retrospective analysis; PS—prospective study; CCT—controlled clinical trial; RCT—randomized controlled trial.
G1—group fresh extraction sockets; G2—group healed sites.
d—days; wk—weeks; mo—months; Mx—maxilla; Md—mandible.
[a] light smokers <10 cig/day; heavy smokers >10 cig/day; extra-heavy smokers >20 cig/day.

Table S2. Quality assessment of the included studies, according to the National Institutes of Health (NIH).

**Study**	**Year**	**Was the study question or objective clearly stated?**	**Was the study population clearly and fully described, including a case definition?**	**Were the cases consecutive?**	**Were the subjects comparable?**	**Was the intervention clearly described?**	**Were the outcome measures clearly defined, valid, reliable, and implemented consistently across all study participants?**	**Was the length of follow-up adequate? [a]**	**Were the statistical methods well-described?**	**Were the results well-described?**	**Total (n/9)**
**Agliardi**	2014	1	1	1	1	1	1	1	1	1	9/9
**Agnini**	2014	1	1	1	1	1	1	1	1	1	9/9
**Aguirre-Zorzano**	2011	1	1	0	1	1	1	1	1	1	8/9
**Aires**	2002	1	1	0	1	1	1	1	0	1	7/9
**Alves**	2010	1	1	1	1	1	1	1	0	1	8/9
**Amato**	2020	1	1	0	1	1	1	1	0	1	7/9
**Andersson**	2015	1	0	1	1	1	1	1	1	1	8/9
**Annibali**	2011	1	1	1	1	1	1	1	1	1	9/9
**Arlin**	2002	1	1	0	1	1	1	1	1	1	8/9
**Arora**	2018	1	1	0	1	1	1	1	1	1	8/9
**Artzi**	2010	1	1	0	1	1	1	1	1	1	8/9
**Atieh**	2013	1	1	0	1	1	1	1	1	1	8/9
**Bae**	2011	1	1	1	1	1	1	1	1	1	9/9
**Barbier**	2012	1	1	1	1	1	1	1	1	1	9/9
**Bettach**	2015	1	1	1	1	1	1	1	1	1	9/9
**Bogaerde**	2010	1	1	1	1	1	1	1	0	1	8/9
**Cakarer**	2014	1	1	1	1	1	1	1	1	1	9/9
**Callan**	2000	1	1	1	1	1	1	1	0	1	8/9
**Cannata**	2017	1	1	1	1	1	1	1	1	1	9/9
**Cannizzaro (1)**	2008	1	1	1	1	1	1	1	1	1	9/9
**Cannizzaro (2)**	2008	1	1	1	1	1	1	1	1	1	9/9
**Cannizzaro (3)**	2011	1	1	1	1	1	1	1	1	1	9/9
**Cannizzaro (4)**	2012	1	1	1	1	1	1	1	1	1	9/9
**Cannizzaro (5)**	2012	1	1	1	1	1	1	1	1	1	9/9

**Cannizzaro (6)**	2015	1	1	1	1	1	1	1	1	1	9/9
**Cannizzaro (7)**	2016	1	1	1	1	1	1	1	1	1	9/9
**Cannizzaro (8)**	2016	1	1	1	1	1	1	1	1	1	9/9
**Cannizzaro (9)**	2016	1	1	1	1	1	1	1	1	1	9/9
**Cannizzaro (10)**	2017	1	1	1	1	1	1	1	1	1	9/9
**Cannizzaro (11)**	2017	1	1	1	1	1	1	1	1	1	9/9
**Cannizzaro (12)**	2018	1	1	1	1	1	1	1	1	1	9/9
**Cannizzaro (13)**	2018	1	1	1	1	1	1	1	1	1	9/9
**Capelli**	2010	1	1	1	1	1	1	1	1	1	9/9
**Carr**	2019	1	1	1	1	1	1	1	1	1	9/9
**Cavallaro**	2011	1	1	1	1	1	1	1	0	1	8/9
**Cercadillo-Ibarguren**	2017	1	1	1	1	1	1	1	1	1	9/9
**Chaushu**	2001	1	1	1	1	1	1	1	0	1	9/9
**Checchi**	2017	1	1	1	1	1	1	1	1	1	9/9
**Ciabattoni**	2017	1	1	1	1	1	1	1	1	1	9/9
**Clementini**	2019	1	1	1	1	1	1	1	1	1	9/9
**Cooper**	2014	1	1	0	1	1	1	1	1	1	8/9
**Correia**	2017	1	1	1	1	1	1	1	1	1	9/9
**Cosyn**	2012	1	1	1	1	1	1	1	1	1	9/9
**Cosyn**	2013	1	1	1	1	1	1	1	1	1	9/9
**Covani**	2012	1	1	1	1	1	1	1	1	1	9/9
**Crespi**	2014	1	1	0	1	1	1	1	1	1	8/9
**Cucchi**	2019	1	1	0	1	1	1	1	1	1	8/9
**Davarpanah**	2002	1	1	0	1	1	1	1	0	1	7/9
**De Bruyn (1)**	2002	1	1	1	1	1	1	1	1	1	9/9
**De Bruyn (2)**	2013	1	1	1	1	1	1	1	0	1	8/9
**Degasperi**	2014	1	1	0	1	1	1	1	0	1	7/9
**Degidi (1)**	2006	1	1	1	1	1	1	1	1	1	9/9
**Degidi (2)**	2007	1	1	0	1	1	1	1	1	1	8/9
**Degidi (3)**	2007	1	1	0	1	1	1	1	1	1	8/9
**Degidi (4)**	2007	1	1	0	1	1	1	1	0	1	7/9
**Degidi (5)**	2012	1	1	0	1	1	1	1	1	1	8/9
**Degidi (6)**	2015	1	1	0	1	1	1	1	1	1	8/9
**Deng**	2010	1	1	0	1	1	1	1	0	1	7/9
**Dhanrajani**	2005	1	1	1	1	1	1	1	0	1	8/9
**Di Alberti**	2012	1	1	1	1	1	1	1	0	1	8/9
**Esposito**	2010	1	1	0	1	1	1	1	1	1	8/9
**Esposito (1)**	2012	1	1	1	1	1	1	1	1	1	9/9
**Esposito (2)**	2015	1	1	1	1	1	1	1	1	1	9/9

**Esposito (3)**	2017	1	1	1	1	1	1	1	1	1	9/9
**Evian**	2004	1	1	1	1	1	1	1	0	1	8/9
**Feher**	2020	1	1	0	1	1	1	1	1	1	8/9
**Felice (1)**	2011	1	1	1	1	1	1	1	1	1	9/9
**Felice (2)**	2014	1	1	0	1	1	1	1	1	1	8/9
**Felice (3)**	2015	1	1	1	1	1	1	1	1	1	9/9
**Felice (4)**	2016	1	1	1	1	1	1	1	1	1	9/9
**Gehrke**	2015	1	1	0	1	1	1	1	0	1	7/9
**Gelb**	2013	1	1	1	1	1	1	1	0	1	8/9
**Gillot**	2011	1	1	1	1	1	1	1	0	1	8/9
**Gillot**	2012	1	1	1	1	1	1	1	0	1	8/9
**Giordano**	2017	1	1	1	1	1	1	1	1	1	9/9
**Gomez-Roman**	1997	1	1	1	1	1	1	1	1	1	9/9
**Grandi**	2013	1	1	1	1	1	1	1	1	1	9/9
**Grandi**	2015	1	1	1	1	1	1	1	1	1	9/9
**Guarnieri**	2020	1	1	1	1	1	1	1	1	1	9/9
**Haas**	1996	1	1	1	1	1	1	1	1	1	9/9
**Horwitz**	2007	1	1	0	1	1	1	1	1	1	8/9
**Huynh-Ba**	2019	1	1	1	1	1	1	1	1	1	9/9
**Irinakis**	2009	1	1	1	1	1	1	1	0	1	8/9
**Jaffin**	2004	1	1	0	1	1	1	1	0	1	7/9
**Ji**	2012	1	1	0	1	1	1	1	0	1	7/9
**Kim**	2017	1	1	0	1	1	1	1	1	1	8/9
**Kohen**	2016	1	1	0	1	1	1	1	1	1	8/9
**Kourtis**	2004	1	1	1	1	1	1	1	0	1	8/9
**Krennmair**	2014	1	1	1	1	1	1	1	1	1	9/9
**Lee (1)**	2018	1	1	0	1	1	1	1	1	0	7/9
**Lee (2)**	2020	1	1	0	1	1	1	1	0	1	7/9
**Leonida**	2012	1	1	0	1	1	1	1	0	1	7/9
**Lindeboom**	2006	1	1	1	1	1	1	1	1	1	9/9
**Locante**	2004	1	1	0	1	1	1	1	0	1	7/9
**Luongo**	2014	1	1	0	1	1	1	1	1	1	8/9
**Malchiodi (1)**	2011	1	0	1	1	1	1	1	1	1	8/9
**Malchiodi (2)**	2016	1	1	0	1	1	1	1	1	1	8/9
**Maló**	2000	1	1	1	1	1	1	1	0	1	8/9
**Maló**	2003	1	1	1	1	1	1	1	0	1	8/9
**Maló**	2003	1	1	1	1	1	1	1	0	1	8/9
**Mangano**	2013	1	1	0	1	1	1	1	0	1	7/9
**Mangano**	2017	1	1	0	1	1	1	1	1	1	8/9
**Meizi**	2014	1	1	1	1	1	1	1	1	1	9/9
**Mensdorff-Pouilly**	1994	1	1	1	1	1	1	1	1	1	9/9
**Mertens**	2011	1	1	0	1	1	1	1	1	1	8/9
**Moraes**	2013	1	1	1	1	1	1	1	0	1	8/9

**Noelken**	2014	1	1	0	1	1	1	1	1	1	8/9
**Norton (1)**	2004	1	1	1	1	1	1	1	0	1	8/9
**Norton (2)**	2017	1	1	1	1	1	1	1	1	1	9/9
**Oliva**	2012	1	1	0	1	1	1	1	0	1	7/9
**Ormianer (1)**	2008	1	1	0	1	1	1	1	1	1	8/9
**Ormianer (2)**	2012	1	1	0	1	1	1	1	1	1	8/9
**Oxby**	2015	1	1	1	1	1	1	1	1	1	9/9
**Palattella**	2008	1	1	0	1	1	1	1	0	1	7/9
**Pellicer-Chover**	2014	1	1	0	1	1	1	1	0		7/9
**Peñarrocha-Diago (1)**	2008	1	1	0	1	1	1	1	1	1	8/9
**Peñarrocha-Diago (2)**	2011	1	1	0	1	1	1	1	0	1	7/9
**Peñarrocha-Diago (3)**	2012	1	1	0	1	1	1	1	0	1	7/9
**Peñarrocha-Oltra (1)**	2012	1	1	0	1	1	1	1	1	1	8/9
**Peñarrocha-Oltra (2)**	2013	1	1	1	1	1	1	1	1	1	9/9
**Perry**	2004	1	1	1	1	1	1	1	0	1	8/9
**Petterson**	2015	1	1	1	1	1	1	1	0	1	8/9
**Piek**	2013	1	1	0	1	1	1	1	1	1	8/9
**Pieri**	2009	1	1	1	1	1	1	1	1	1	9/9
**Polizzi (1)**	2000	1	0	1	1	1	1	1	0	1	7/9
**Polizzi (2)**	2015	1	1	1	1	1	1	1	1	1	9/9
**Pozzi (1)**	2014	1	1	1	1	1	1	1	1	1	9/9
**Pozzi (2)**	2015	1	1	1	1	1	1	1	1	1	9/9
**Pozzi (3)**	2016	1	1	1	1	1	1	1	1	1	9/9
**Prati (1)**	2020	1	1	1	1	1	1	1	1	1	9/9
**Prati (2)**	2020	1	1	1	1	1	1	1	1	1	9/9
**Raes (1)**	2012	1	1	0	1	1	1	1	1	1	8/9
**Raes (2)**	2013	1	1	1	1	1	1	1	1	1	9/9
**Raes (3)**	2017	1	1	0	1	1	1	1	1	1	8/9
**Raes (4)**	2018	1	1	0	1	1	1	1	1	1	8/9
**Ribeiro**	2008	1	1	1	1	1	1	1	0	1	8/9
**Rodrigo**	2012	1	1	1	1	1	1	1	1	1	9/9
**Romanos (1)**	2012	1	1	0	1	1	1	1	0	1	7/9
**Romanos (2)**	2014	1	1	0	1	1	1	1	0	1	7/9
**Romanos (3)**	2014	1	1	0	1	1	1	1	0	1	7/9
**Saridakis**	2018	1	1	0	1	1	1	1	1	1	8/9
**Sato**	2014	1	1	0	1	1	1	1	0	1	7/9
**Schiegnitz**	2016	1	1	0	1	1	1	1	1	1	8/9
**Schlee**	2014	1	1	0	1	1	1	1	1	1	8/9

**Schoenbaum**	2021	1	1	0	1	1	1	1	1	1	8/9
**Sennerby**	2008	1	1	1	1	1	1	1	0	1	8/9
**Siciliano**	2009	1	1	1	1	1	1	1	1	1	9/9
**Siebers**	2010	1	1	0	1	1	1	1	0	1	7/9
**Swart**	2014	1	1	1	1	1	1	1	0	1	8/9
**Tallarico (1)**	2016	1	1	1	1	1	1	1	1	1	9/9
**Tallarico (2)**	2017	1	1	1	1	1	1	1	1	1	9/9
**Testori**	2014	1	1	1	1	1	1	1	1	1	9/9
**Thome (1)**	2020	1	1	1	1	1	1	1	1	1	9/9
**Thome (2)**	2021	1	1	0	1	1	1	1	1	1	8/9
**Tonetti**	2017	1	1	1	1	1	1	1	1	1	9/9
**Urdaneta**	2012	1	1	0	1	1	1	1	1	1	8/9
**van Kesteren**	2010	1	1	0	1	1	1	1	1	1	8/9
**Vandeweghe**	2012	1	1	1	1	1	1	1	0	1	8/9
**Walton**	2016	1	1	1	1	1	1	1	1	0	9/9
**Watzek**	1995	1	1	0	1	1	1	1	0	1	7/9
**Wolfinger**	2003	1	1	1	1	1	1	1	0	1	8/9
**Zafiropoulos**	2010	1	1	0	1	1	1	1	1	1	8/9
**Zumstein**	2016	1	1	1	1	1	1	1	1	1	9/9

[a] 3 months of follow-up was considered to be of adequate length.
